# Bioactive Supermolecules: The Promising Assemblies Presented in Traditional Chinese Medicine

**DOI:** 10.2174/0115672018405057251201120940

**Published:** 2026-04-30

**Authors:** Zhongrui Li, Yu Zheng, Haoyue Ge, Lei Wang, Chao Han

**Affiliations:** 1 Department of Rehabilitation, College of Acupuncture and Moxibustion and Massage Health Preservation and Rehabilitation, Nanjing University of Chinese Medicine, Nanjing, No. 138 Xianlin Avenue, Nanjing 210023, PR China;; 2 State Key Laboratory of Natural Medicines, Jiangsu Key Laboratory of Bioactive Natural Product Research, School of Traditional Chinese Pharmacy, China Pharmaceutical University, 639 Longmian Avenue, Nanjing 211198, PR China

**Keywords:** Traditional Chinese medicine, self-assembled supramolecule, decoction, Chinese carbonized drugs, fresh herbs, enhanced efficiency

## Abstract

Rooted in Eastern philosophy and culture, traditional Chinese medicine (TCM) has safeguarded human health for millennia and remains a vital source for novel drug discovery. Notably, supramolecular nanostructures, spontaneously formed through non-covalent interactions among multiple bioactive components, have been identified in TCM and show significant therapeutic potential. These supramolecules exhibit unique therapeutic advantages by improving the aqueous solubility of lipophilic bioactive constituents and demonstrating synergistic pharmacological effects with reduced toxicity profiles. This review consolidates recent advances in three categories of supramolecular nanoplatforms, focusing on: (1) self-assembled nanoaggregates from TCM decoction, (2) self-assembled carbonized nanoarchitectures from Chinese carbonized drugs, and (3) self-assembled extracellular vesicle-like nanoparticles from Chinese fresh herbs. This paper mainly introduces the nanoplatforms’ morphology, self-assembled mechanisms, and pharmacological activities of these supramolecules. Bioactive supramolecular assemblies derived from traditional Chinese medicine (TCM) offer novel insights into TCM’s material foundation and present potential therapeutic alternatives for disease treatment, thereby advancing TCM modernization efforts.

## 1 INTRODUCTION

Traditional Chinese medicine (TCM) is China’s extensive and profound traditional culture and advanced medicine. It has sustained population health through centuries of clinical practice. After thousands of years of continuous summary and induction of disease understanding and drug experience, it gradually forms and improves the theory of TCM with a unique theoretical system, which fully reflects the characteristics and advantages of Chinese philosophy, history and culture, and lifestyle. Within TCM’s theoretical framework, disease pathogenesis is attributed to dysregulation in the Yin-Yang energetic system [1, 2]. Clinical management, therefore, centers on recalibrating the harmonious interaction between these complementary forces. TCM therapy highlights the holistic view and syndrome differentiation, following the fundamental principle of building up the body’s resistance and eliminating evils. Thus, TCM holds great potential for prevention and treatment of multiple illnesses, such as autoimmune disorders, cardiovascular diseases, and serious diseases of unknown etiology [3-5]. With advances in modern analytical techniques, combined with pharmacokinetics and pharmacodynamics studies, an increasing number of active ingredients in Chinese herbs and compound formulations have been identified [6, 7]. However, each Chinese herb represents a complex system, encompassing a wide variety of chemical components and primary metabolites with diverse structures. The composition of clinically used TCM formulas is even more intricate. Therefore, it remains challenging to characterize the therapeutic constituents of TCM based solely on isolated active ingredients. A deeper understanding requires further investigation into the interactions, existence forms, and phase states of the various components within TCM.

Supramolecular nanoparticles, which spontaneously organize through multiple non-covalent associations such as electrostatic interaction, hydrogen bond, π-π stacking, and coordination interactions, have shown significant potential as materials for disease therapy [8, 9]. These supramolecules exhibit unique therapeutic advantages by improving the aqueous solubility of lipophilic bioactive constituents and demonstrating synergistic pharmacological effects with reduced toxicity profiles. In addition, the supramolecular nanoplatforms assembled without exotic non-therapeutic carriers exhibit enhanced safety and minimal toxicity in comparison to conventional nanodrugs. Recently, an accumulating body of research indicates that complex physical and chemical interactions always inevitably occur during the decoction process of TCM to generate self-assembled nanoaggregates, such as nanospheres, nanomicelles, nanovesicles and nanofibers [10, 11]. The process of nanoaggregates formation contains the scientific connotation of TCM compatibility, which not only reduces the toxicity and side effects but also complements each other to achieve a synergistic effect. Similarly, self-assembled carbonized nanoarchitectures are discovered in the extracts of Chinese carbonized drugs, which are widely utilized as adjuvant therapies for hemorrhagic disorders [12, 13]. The pharmacological activities of carbonized nanoarchitectures in charcoal drugs have attracted attention and praise from many scholars. As for Chinese fresh herbs, self-assembled extracellular vesicle-like nanoparticles are found to be nanoscale membranous vesicles isolated from herb-derived fresh juices. These bioactive nanoparticles comprise lipid bilayers encapsulating diverse biomolecules, including proteins, polysaccharides, genetic material and various metabolites [14-16]. These extracellular vesicles have been confirmed as natural therapeutic agents to show unique functions and functional drug carriers to improve their effects.

**Fig. (1):**
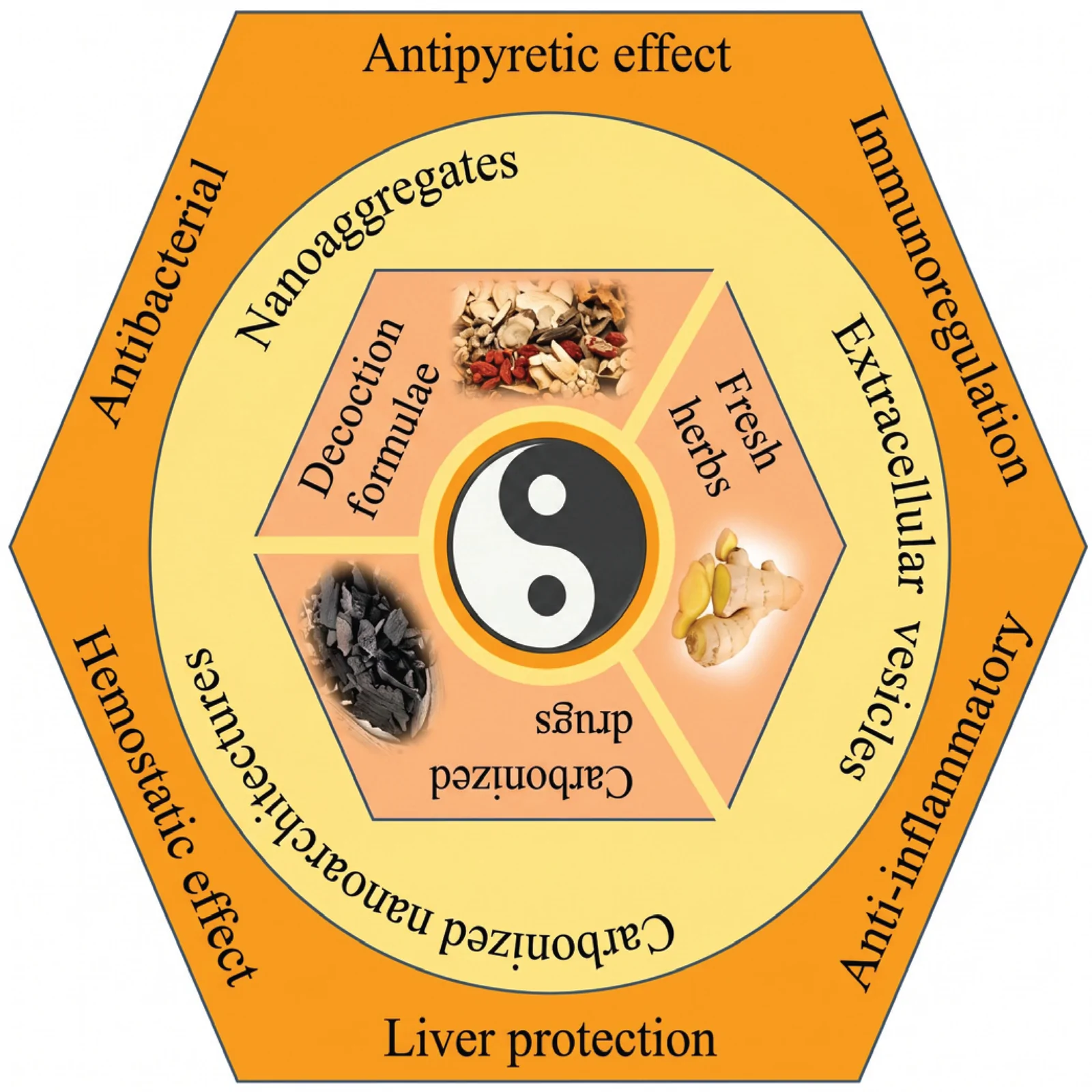
Self-assembled supramolecule nanoplatforms from TCMs and their bioactivity.

Thus, the rapid development of bioactive self-assembled nanoplatforms derived from traditional Chinese medicines warrants systematic analysis. This review consolidates recent advances in three categories of supramolecular nanoplatforms, focus on (Fig. **1**): (i) self-assembled nanoaggregates from decoction of TCM; (ii) self-assembled carbonized nanoarchitectures from Chinese carbonized drugs; (iii) self-assembled extracellular vesicle-like nanoparticles from Chinese fresh herbs. An emphasis will be placed on the sources, traditional efficacy, nanoplatforms morphology, self-assembled mechanisms, and modern pharmacological activities. Throughout this review, we argue that these self-assembled nanostructures represent a crucial, yet underexplored, material basis of TCM efficacy, providing a novel nano-scale perspective that bridges traditional philosophy and modern science. Finally, a forward-looking analysis of opportunities and challenges is presented for biologically active, TCM-derived self-assembled nanoplatforms.

## 2 METHODOLOGY

The data reported in this research come from various electronic databases (ScienceDirect, Scopus, Google Scholar, Web of Science, SciFinder, Wiley Online, SpringerLink, PubMed, and CNKI). Search terms were tailored to capture studies on TCM-derived self-assembled supramolecular nanoplatforms, with adjustments for database-specific indexing rules. Core terms included: TCM source-related: “Traditional Chinese Medicine”, “TCM decoction”, “Chinese carbonized drugs”, “fresh Chinese herbs”, “herbal formulae”. Nanoplatform-related: “self-assembled supramolecules”, “nanoaggregates”, “carbonized nanoarchitectures”, “extracellular vesicle-like nanoparticles”, “natural nanoparticles”. Mechanism and activity-related: “self-assembly mechanism”, “non-covalent interactions”, “pharmacological activity”, “antibacterial”, “anti-inflammatory”, “hemostatic”, “liver protection”. Terms were combined using Boolean operators (“AND”, “OR”) to refine searches (*e.g*., “(TCM decoction OR Chinese carbonized drugs) AND (self-assembled nanoaggregates OR carbonized nanoarchitectures)”).

Studies were included if they met the following criteria: Focused on self-assembled supramolecular nanoplatforms derived from TCM, specifically: (1) Self-assembled nanoaggregates formed in TCM decoctions; (2) Self-assembled carbonized nanoarchitectures isolated from Chinese carbonized drugs; (3) Self-assembled extracellular vesicle-like nanoparticles extracted from fresh Chinese herbs. Provided experimental data on nanoplatform characterization (*e.g*., size, morphology, chemical composition), self-assembly mechanisms (*e.g*., electrostatic interactions, hydrogen bonding, π-π stacking), or *in vitro*/*in vivo* pharmacological activities. Published as peer-reviewed journal articles with full-text availability in English or Chinese (non-English articles were translated to English for analysis when necessary). Included original experimental data (*in vitro* cell studies, *in vivo* animal models, or preclinical evaluations).

Studies were excluded if They were editorials, conference abstracts, or book chapters without original experimental data. They focused on synthetic nanocarriers not involving natural self-assembly processes in TCM. They lacked essential information on nanoplatform characterization, mechanism, or pharmacological outcomes. They were duplicate publications (only the most comprehensive study was retained to avoid redundancy).

Data from included studies were synthesized qualitatively due to the heterogeneity of nanoplatform types, TCM sources, and experimental models. Key findings were categorized by nanoplatform type (nanoaggregates, carbonized nanoarchitectures, extracellular vesicle-like nanoparticles) and summarized in structured tables to visualize characteristics, mechanisms, and pharmacological activities. Mechanistic insights into self-assembly processes and structure-activity relationships were integrated to identify common principles, research gaps, and translational potential, guiding the narrative structure of this review.

## 3 SELF-ASSEMBLED NANOAGGREGATES FROM DECOCTION OF TCM

Decoction is the liquid part after decocting Chinese herbs with water, which is the earliest and most widely used dosage form in TCM clinics [17, 18]. This dosage form possesses the advantages of fast absorption, quick action and high bioavailability. Meanwhile, it can be conveniently used to add or subtract herbs according to the differences of patients' syndromes and can also meet the needs of TCM treatment based on syndrome differentiation. In fact, decoction is a complex and dispersed system. A large number of primary metabolites (such as polypeptides, glycans, phospholipids, and nucleotides), secondary metabolites (including a variety of small natural molecules) and other portions of plant cells can be dissolved in water solution during the decocting process [19]. These substances undergo complex chemical and physical changes to form different nanoaggregates (NAs) in the decoction. Notably, the formation of NAs through interactions among herbal components aligns well with the TCM principles of holistic treatment and syndrome differentiation.

### 3.1 Decoction of Herbal Formulae

**Fig. (2):**
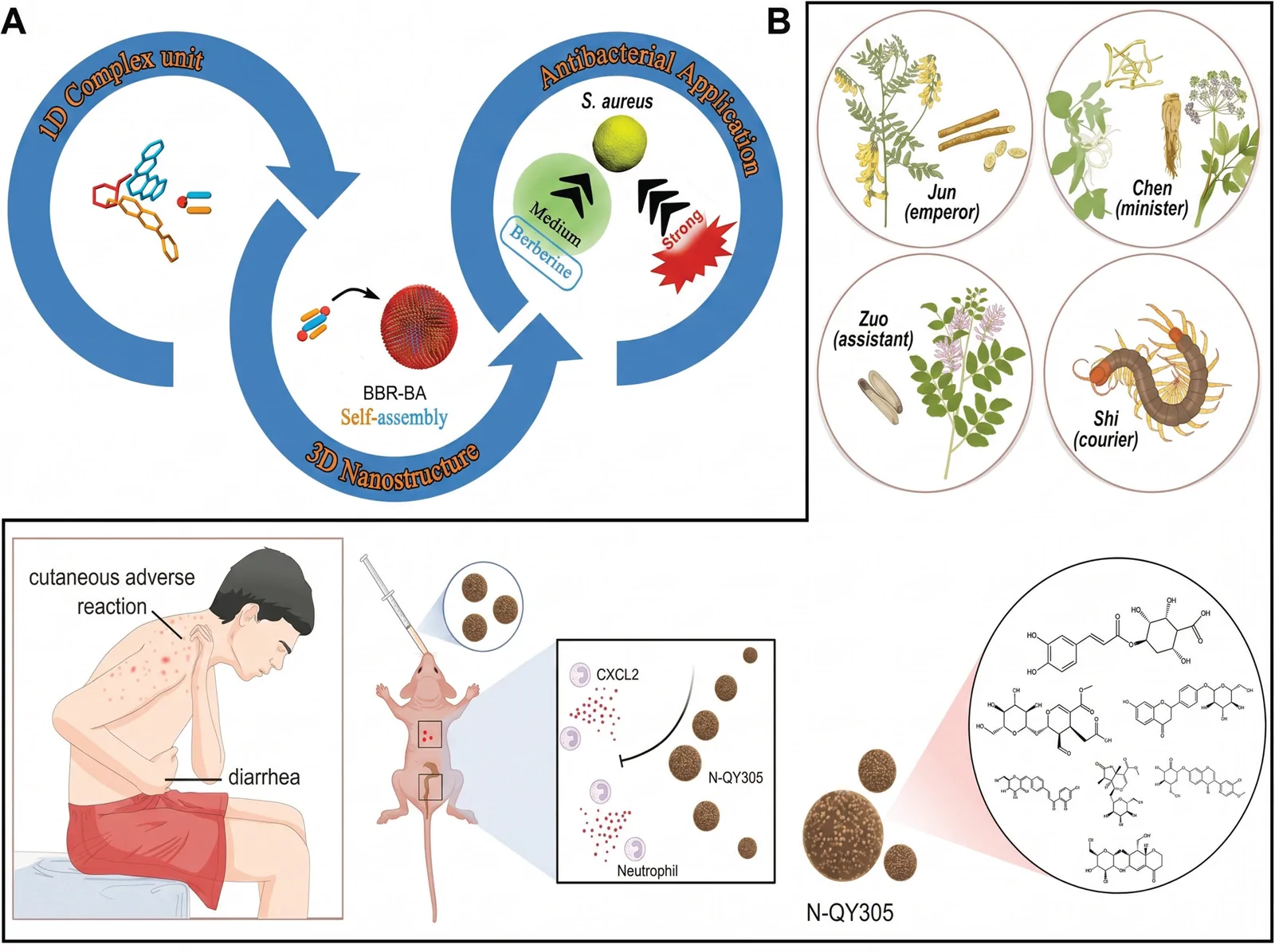
(**A**) Antibacterial nanoaggregates formed through self-assembly of BBR-BA. Reproduced with permission from Ref. [24]. Copyright 2019, American Chemical Society; (**B**) Nanoparticulate assembly of QY305 (N-QY305) attenuating drug-induced dermatological and enteric adverse events. Reproduced with permission from Ref. [27]. Copyright 2023, John Wiley and Sons.

Huang-Lian-Jie-Du decoction (HLJDD), a traditional herbal formula, combines four key medicinal herbs: Coptidis Rhizoma, Scutellariae Radix, Phellodendri Chinensis Cortex, and Gardeniae Fructus in a 3:2:2:3 ratio. Known for its heat-clearing and detoxifying properties, this remedy is widely applied in clinical practice to manage various conditions, including malignancies, renal impairment, metabolic disorders like diabetes, and ischemic stroke [20]. Lei’s group [21-23] found the contents of berberine (BBR) and baicalin (BA) were significantly higher in NAs than in the supernatant from HLJDD, and then the main source of HLJDD-NAs was Coptidis Rhizoma and Scutellarine Radix. The formation of BBR-BA nanoparticles with a binding ratio of 1:1 might be derived from acid-base neutralization as well as hydrophobic forces and electrostatic attraction. During self-assembly, BBR (isoquinoline alkaloid) and BA (flavonoid glycoside) initially formed linear molecular arrangements, which then spontaneously assembled into higher-order nanoarchitectures with three-dimensional morphological features (Fig. **2A**). Notably, these nanoparticles were reported to demonstrated markedly improved antibacterial effects targeting *Staphylococcus aureus* [24]. These nanoparticles displayed good biocompatibility and stronger affinity to bacteria due to the hydrophilic glycosyl toward the outside, thereby leading to a decrease in bacterial biofilm and the collapse of the bacterial population. Furthermore, BBR-BA nanoparticles showed stronger synergistic therapeutic effects in a mouse model of diarrhea-dominant irritable bowel syndrome (IBS-D) compared to a crude blend of BBR and BA [25], yet the long-term safety and dose-response relationship of this nanoformulation remains to be systematically investigated.

Similar self-assembly phenomena and enhanced efficacy have been observed in other herbal formulae. Qian *et al* [26] developed a supramolecular complex of BBR and chlorogenic acid (CGA), matching the proportional composition of these two compounds found in the naturally self-organized bioactive constituents derived from Huanglian Wumei decoction (HLWMD). The BBR-CGA nanoparticles were also reported to demonstrate superior suppression of inflammatory cytokine release and LPS-triggered cell death compared to unbound compounds, primarily through blocking NF-κB p65 activation and the caspase-11-dependent alternative pyroptosis pathway. In parallel, Zhang *et al* [27] isolated N-QY305, a nanoscale fraction from the classic formula T-QY305 (used for dermatological and digestive disorders), which exhibited uniform spherical morphology and contained 13 bioactive compounds. This nano-preparation was found to show superior therapeutic outcomes relative to the crude extract in alleviating epidermal growth factor receptor inhibitors (EGFRIs)-related skin toxicity and gastrointestinal symptoms, particularly through regulation of neutrophil accumulation in cutaneous and intestinal tissues (Fig. **2B**).

Bai-Hu decoction (BHD), another ancient clinical formula dosage form in China, demonstrates therapeutic efficacy against influenza, pneumonia and acute suppurative tonsillitis [28]. BHD contains three herbs (Anemarrhenae Rhizoma, Glycyrrhiza Radix et Rhizoma [GR], and japonica rice) and one mineral drug (gypsum). NAs derived from BHD showed diameters of 100 nm and involved multiple elements such as C, O, Mg, Si, and Ca [29, 30]. Different herbal components contributed uniquely to nanoparticle assembly. Japonica rice provided colloidal macromolecule (starches), while gypsum contained ions (Si, Na, Ca, and Mn) to stabilize the nano-phase by charge effects. Notably, bioactive constituents in GR (including liquiritin, glycyrrhizic acid, and glycyrrhizin) likely functioned as natural surfactants to increase solubilization. Anemarrhenae Rhizoma offered antipyretic entities (neomangiferin and mangiferin) during the nano-phase formation. Ultimately, BHD-NAs were reported to show a prominent antipyretic effect through suppression of key inflammatory markers including interleukin-1β (IL-1β), transient receptor potential vanilloid 4 (TRPV4), nuclear factor-kappa B (NF-κB), and tumor necrosis factor-alpha (TNF-α).

**Fig. (3):**
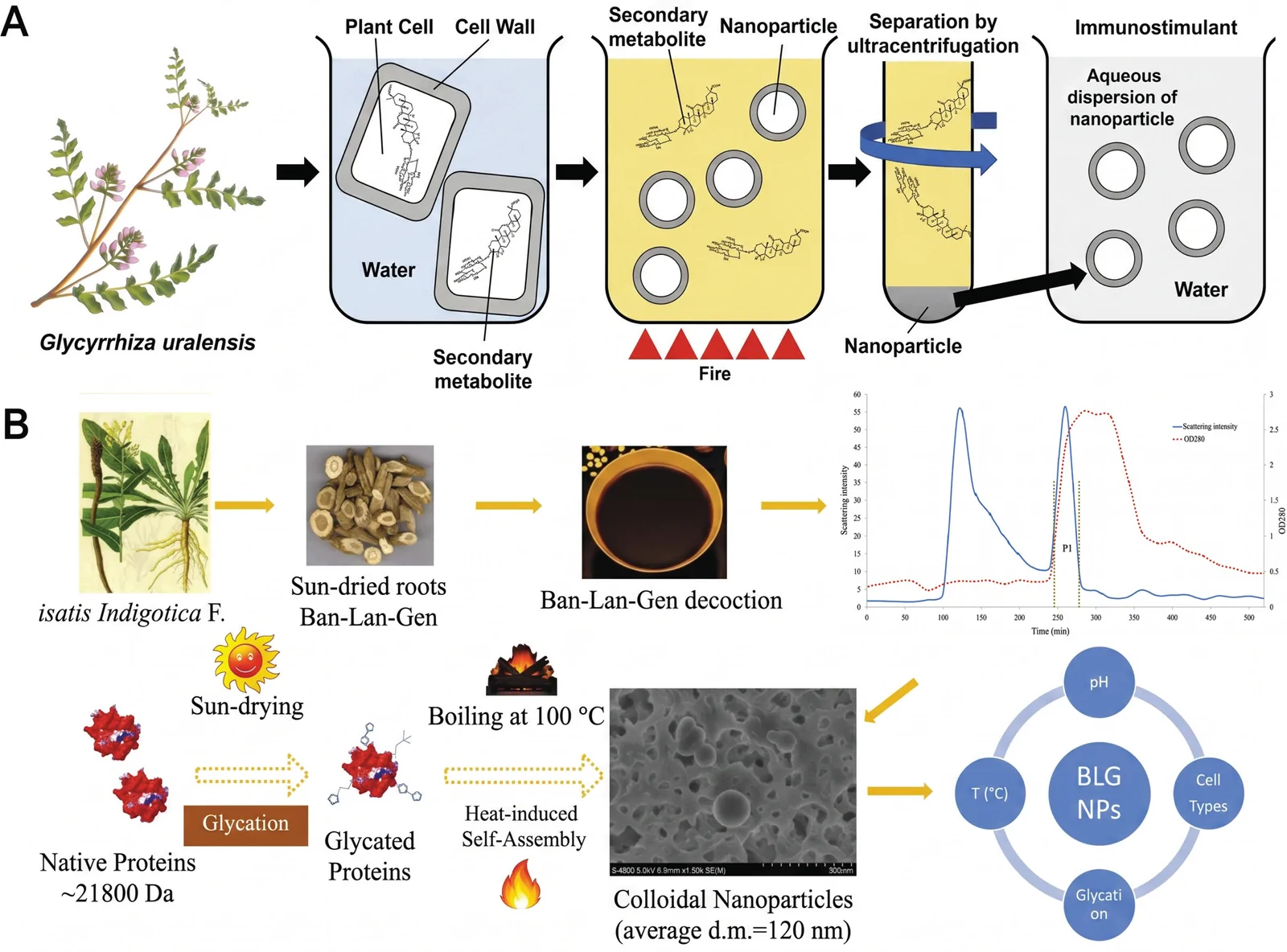
(**A**) Sugar-based nanoaggregates existing in GRD and their immunostimulant effect. Reproduced with permission from Ref. [57]. Copyright 2018, Elsevier Inc.; (**B**) Colloidal nanoparticles as intelligent nanoassemblies of a boiling-stable protein isolated from IRD. Reproduced with permission from Ref. [72]. Copyright 2017, Elsevier Inc.

With a documented clinical history spanning two millennia, Ma-Xing-Shi-Gan decoction (MXSGD) is characterized by four principal therapeutic actions in traditional Chinese medicine: lung heat clearance, asthma relief, cough suppression, and phlegm resolution [31, 32]. *Ephedrae Herba* is deemed as the principal ingredient in MXSGD, while the other three ingredients (Armeniacae Semen Amarum, GR, and gypsum) are also important components. Ke’s group [33, 34] successfully separated NAs from MXSGD using size-exclusion chromatography (Fig. **3A**). MXSGD-NAs (50-200 nm diameter) predominantly contained ephedrine (99.7%) and pseudoephedrine (95.5%) as principal components. These NAs were found to show anti-influenza A virus activity *in vitro*, which might be related to the content of ephedrine and pseudoephedrine. Subsequently, Zhao *et al* [35] successfully isolated MXSGD-derived nanoparticles through sequential ultracentrifugation and dialysis techniques, achieving particles of 400-500 nm diameter. Notably, these nanoparticles shared compositional profiles with gypsum-containing decoctions and demonstrated comparable fever-reducing properties, revealing the material basis for gypsum’s essential role in this classical formulation.

Ge-Gen-Qin-Lian decoction (GGQLD), a classical formula dating back to the Eastern Han Dynasty (25-220 AD), is traditionally prescribed for exterior heat syndrome. This formulation integrates four botanical components: Puerariae Radix, Scutellariae Radix, Coptidis Rhizoma, and GR. Contemporary research confirms its therapeutic applications in managing non-insulin dependent diabetes mellitus and inflammatory bowel disorders [36]. GGQLD-NAs were reported to enhance the bioavailability of BA, upregulate antioxidant enzymes, protect pancreatic 𝛽-cells from streptozotocin-induced oxidation and recover insulin secretion [37, 38]. Besides, GGQLD-NAs showed little toxicity on 𝛽-cells and colonic epithelial cells. GGQLD-NAs might unveil new dimensions of phytotherapeutic synergy in diabetes treatment, particularly regarding the integrated bioactivity of complex herbal systems.

Gancao Ganjiang decoction (GGD), a combination of *Glycyrrhiza uralensis* and *Zingiber officinale*, is a classic TCM formula known for its warming and restorative properties targeting the spleen and lung systems. Clinical studies suggest its efficacy in managing conditions like lung atrophy, respiratory distress, and symptoms associated with malaria [39]. He *et al* [40] employed differential centrifugation to isolate GGD-NAs. Comparative analysis of the individual herbal extracts *versus* the combined formulation revealed a substantial alteration in 6-gingerol content following compatibility. The solubility of 6-gingerol was notably enhanced in the composite GGD system relative to the single-ingredient Ganjiang decoction. This was due to the spontaneous nanoparticle formation *via* molecular self-assembly between hydrophilic glycyrrhizic acid and 6-gingerol, thereby enhancing the solubility of the latter.

Xie-Bai-San Decoction (XBSD), with origins tracing to Southern Song medical literature, remains clinically relevant for heat-induced pulmonary conditions spanning from acute nasopharyngitis to pediatric lower respiratory tract infections, all manifesting traditional “fei re” syndrome patterns. XBSD incorporates a quadripartite botanical composition: Cortex Mori, Cortex Lycii, GR, and Japonica rice. Nie *et al* [41] successfully isolated XBSD-NAs (104.53 ± 2.1 nm diameter, 0.27 PDI, ζ-potential -5.14 mV). Most of the eight active compounds from XBSD were found to be present in XBSD-NAs. The study showed that Kukoamine B spontaneously forms nanoparticle complexes by interacting with Mulberroside A or Liquiritin. Pharmacokinetic studies revealed a marked improvement in the active compounds’ bioavailability following XBSD-NAs administration, suggesting that the natural nanoparticles in the decoction play a crucial role in its therapeutic effects.

Huangqi Gegen decoction (HGD), a traditional Chinese herbal preparation containing Astragali Radix and Puerariae Radix as its primary components, has been clinically applied since its documentation in the 17^th^ century medical text Zhengzhi Huibu. This formulation demonstrates significant efficacy in thrombosis management. Astragalus polysaccharide is reported to improve the aqueous solubility of puerarin through the formation of self-assembled nanoscale particles [42]. This structural modification facilitates greater uptake in the intestines and boosts bioavailability when administered orally. As a result, the HMGB1-mediated NF-κB and NLRP3 signaling cascade is suppressed, leading to an amplified therapeutic effect against thrombosis.

Naoluo Xintong decoction (NXD), a traditional Chinese medicine formulation with demonstrated effects in acute cerebral ischemia [43], comprises seven active components: Astragali Radix, Chuanxiong Rhizoma, Notoginseng Radix et Rhizoma, Gastrodiae Rhizoma, Carthami Flos, Angelicae Sinensis Radix, and Scolopendra. Astragali Radix serves as the principal therapeutic agent, with complementary herbs exhibiting synergistic bioactivity through coordinated pharmacological actions. Zhao *et al* [44] isolated and characterized NAs from NXD, and studied their brain protection effect using the middle cerebral artery occlusion rat model. Their analysis revealed NXD-NAs (200~400 nm) primarily consisted of polysaccharides, proteins, and saponins (ginsenoside Rg1, Rb1, and astragaloside IV). NXD-NAs were found to reduce oxidative stress and improve nerve function, which was closely related to the loaded saponins. Hasson *et al* [45] purified NAs from Shi-Quan-Da-Bu decoction (SQDBD) and studied their potent immunostimulatory activity. This decoction is clinically used to enhance patients’ immunologic functions [46]. They discovered that larger SQDBD-NAs (about 100 nm) exhibited modest macrophage-stimulatory activity, while smaller SQDBD-NAs (<10 nm) showed higher potency. Notably, the mechanism underlying this size-dependent activity (*e.g*., differences in cellular uptake efficiency or ligand presentation) was not fully explored, limiting the translatability of these findings. Yunnan Baiyao (YB) has accumulated more than a hundred years of documented use for diverse therapeutic indications across multiple body systems [47]. Lenaghan *et al* [48] measured the nano-scale structures from YB by using atomic force microscopy. Nanostructural analysis identified well-defined fibrous components in YB decoction, featuring invariant 25 nm diameters and substantial length polymorphism (86-726 nm). These nanofibers could induce platelet aggregation to seal wounds due to their unique adhesive and structural properties.

In general, the inherent components could form supramolecular structure through self-organization, self-recognition and self-assembly during the process of herbal formulae decoction. The existence form and physiological activities of formulae decoction supramolecules provided new ideas for study of the material basis of TCM.

### 3.2 Decoction of Single Chinese drug

Similar to herbal formulae, NAs are also generated during decocting process of single Chinese drug and frequently coprecipitate with active ingredients. Coptidis Rhizoma, a famous Chinese herb, is derived from the dried rhizomes of *Coptis chinensis* Franch. It has the traditional function of clearing heat and drying dampness, reducing fire and detoxifying. BBR is the main active ingredient in Coptidis Rhizoma, which has been proven to treat hypertension, hyperlipidemia, and myocarditis [49-51]. NAs, derived from Coptidis Rhizoma decoction (CRD), were mainly composed of Coptidis Rhizoma polysaccharides and featured a 100 nm size and were negatively charged [52-54]. CRD-NAs are reported to enhance BBR absorption in intestinal epithelial cells in a charge-dependent manner, *via* active transport and endocytosis, by modulating tight junction. HoweverB). However, the long-term impact of this tight junction modulation on intestinal barrier integrity (*e.g*., increased permeability to toxins) has not been evaluated. On the other hand, CRD-NAs could also promote BBR absorption through activating the Peyer’s patches-associated immunity. These CRD-NAs suppressed immune activation in Peyer’s patches, downregulating tight junction protein expression. Together, CRD-NAs actively modulate gut barrier function *via* immune activity in Peyer’s patches and T-cell specialization. Further research [55] showed proteinaceous module in CRD-NAs was crucial to decreasing acute oral toxicity of CRD. Proteinaceous CRD-NAs could be used as drug carriers to increase plasma exposure and biological activities of BBR.

GR (*Glycyrrhiza uralensis* Fisch. rhizome) is commonly included in numerous TCM formulations, serving a key function in harmonizing and balancing the properties of companion herbs based on TCM principles. Zhou *et al* [56] isolated NAs from GR decoction (GRD) using sequential chromatography. The self-assembled GRD-NAs were determined to have 23 protein molecules. These NAs exhibited low cellular toxicity and demonstrated penetration ability across four cells (MDCK, L-02, HepG2, and Caco2). Furthermore, they served as drug carriers to encapsulate insoluble astragaloside IV, thereby enhancing its bioavailability. Iitsuka *et al* [57] prepared sugar-based GRD-NAs with a structure of being formed by a polysaccharide and studied their immunostimulant effect (Fig. **3A**). The carbohydrate-rich nanoparticles enhanced the expression of pro-inflammatory IL-6 in macrophages. In addition, glycyrrhiza polysaccharides were also utilized to assemble a nanocomposite with other bioactive components to enhance the bioactivity [58], which were promising templates for design of novel biomaterials in industry and medicine. Li *et al* [59] prepared and explored the NAs formation in the decocting process of GR-Coptidis Rhizoma herb pair. They found glycyrrhiza protein from GR and BBR from Coptidis Rhizoma could form spherical supramolecular particles by electrostatic attraction and hydrophobic interaction. These particles were reported to significantly increase the antimicrobial activity of BBR. Self-assembled NAs also existed in the decoction of GR-Paeoniae Radix Alba herb pair [60, 61]. These NAs, containing paeoniflorin and benzoylpaeoniflorin from Paeoniae Radix Alba and glycyrrhizic acid from GR, significantly enhanced the absorption of these active small molecules, thereby increasing bioavailability. Interestingly, self-assembled NAs from the herb pair GR-Aconiti Radix could reduce the toxicity of Aconiti Radix [62]. Glycyrrhiza proteins from GR interacted with aconitine from Aconiti Radix to form aggregates, thereby reducing its toxicity.

Puerariae Lobatae Radix, the root of Pueraria lobata, has been used to treat angina pectoris, hypertension, influenza, and neck stiffness [63]. Hu’s group [64, 65] demonstrated that naturally occurring NAs in Puerariae Lobatae Radix decoction contain bioactive molecules including puerarin, daidzein, daidzin, and genistein. Mechanistically, these NAs form through self-assembly, cross-molecular association, or metabolite-facilitated aggregation. Their research further elucidated the correlation between NA particle size, intestinal bioavailability, and the therapeutic efficacy of decoctions prepared at varying concentrations. Wang’s group [66, 67] simulated the process of Pseudostellariae Radix decoction and constructed Pseudostellariae Radix protein-based NAs with loading curcumin. These NAs exhibited stronger reducing power, better thermal stability and light stability than free curcumin. Meanwhile, internalization of curcumin could be easily conducted through macropinocytosis and clathrin-mediated endocytosis. Next, Pseudostellariae Radix protein-polysaccharide could also be used as a nanocarrier to load chemotherapeutic doxorubicin [68]. The nanocarriers exhibited pH-dependent drug release and enhanced doxorubicin potency against HepG2 cells by suppressing P-glycoprotein activity. However, the study did not evaluate the toxicity of these nanocarriers in normal liver cells (*e.g*., L-02 cells), which is critical for assessing their safety profile as chemotherapeutic delivery systems. Similar to Pseudostellariae Radix protein, proteins from Armeniacae Semen Amarum [69] or *Phaseolus angularis* [70] could be formed to NAs through rearrangement of the disulfide bond during the decoction process. The thermally aggregated NAs (average diameter: 92 nm) demonstrated low cytotoxicity toward L-02 and MDCK cells, while exhibiting potential as naturally derived delivery vehicles for hydrophobic compounds.

Isatidis Radix, the dried roots of *Isatis indigotica* Fort., has been clinically applied for centuries against infection-related and inflammatory conditions, including viral influenza, hepatic disorders, and type B brain inflammation [71]. Isatidis Radix decoction (IRD) was found to contain abundant natural nanoassemblies (NAs). The decoction process likely induced Maillard reactions, producing non-enzymatic glycated proteins with enhanced structural stability and solubility (Fig. **3B**). IRD-NAs, averaging 120 nm in diameter, exhibited responsiveness to environmental pH and thermal variations [72]. Further analysis confirmed glycated proteins within these NAs, demonstrating stimulatory effects on L-02 cell proliferation while inhibiting growth in Hep-G2 and HeLa-229 cell lines. However, Gao *et al* [73] isolated NAs with a high content of polysaccharides from IRD. These NAs exhibited potent inhibition of H1N1 virus replication, with both antiviral efficacy and cytotoxicity toward MDCK cells showing size-dependent characteristics. Wang *et al* [74] confirmed the self-assembled NAs could be formed when the aqueous extract of *Rabdosia rubescens* leaves was dissolved in water. The antithrombotic utility of *R. rubescens* extract might be related to the formed NAs with concentration-dependently inhibiting P-selectin release.

Rhodiolae Crenulatae Radix et Rhizoma (RCRR), the dried roots and rhizomes of *Rhodiola crenulata*, is first described in Shennong’s Herbal Classic for invigorating Qi, promoting blood circulation, smoothing pulse and relieving asthma. Modern pharmacological research showed that it could clear free radicals and assist the improvement of regulatory immunity [75]. Jiang’ s group [76, 77] discovered the NAs formation from RCRR decoction. These nanoparticles demonstrated strong therapeutic activity against fibrosis and inflammation, containing lipid components including sphinganine (d22:0) and phosphocholine species (18:0/18:2, 16:0/18:2), along with hydrophobic compounds, proteins, and small RNAs. Thermal extraction promoted joint aggregation of sRNAs with lipids, achieving the NA micellization threshold. RCRR-NAs provided a novel therapeutic strategy for delivering plant sRNAs. In conclusion, the self-assembly of natural nanoaggregates (NAs) offers an innovative paradigm to elucidate the pharmacological mechanisms of individual Chinese herbal decoctions, serving as a bioavailability booster for phytochemical delivery. While the self-assembly within single herbs is intriguing, the combination of herbs in formulae, guided by TCM compatibility theory, leads to even more complex and emergent nanostructures, as discussed in the previous section on herbal formulae.

### 3.3 Critical Challenges and Future Perspectives

Despite the promising potential of NAs, several challenges impede their further investigation and application. Firstly, the formation mechanisms are often deduced rather than rigorously proven. *In situ* characterization techniques, such as small-angle X-ray scattering (SAXS) or cryo-electron microscopy during the decoction process, are needed to dynamically capture the self-assembly process. Secondly, the standardisation of preparation and isolation protocols is lacking. Factors like herb origin, decoction time, temperature, and water quality can significantly influence NA characteristics, making reproducibility a concern. Thirdly, most pharmacological studies remain preliminary. The relative contribution of the NAs themselves versus the released free molecules to the overall efficacy is poorly delineated. Future research should employ more sophisticated experimental designs, such as constructing NA-deficient decoctions for comparative efficacy studies, to unequivocally establish their functional role.

## 4 SELF-ASSEMBLED CARBONIZED NANOARCHITECTURES FROM CHINESE CARBONIZED DRUGS

Chinese carbonized drugs constitute a unique category in TCM. These specially processed agents have been clinically employed for over 2000 years, showing proven efficacy against various hemorrhagic conditions [78]. Carbonized drugs are processed by carbonizing medicinal materials at a high temperature, following traditional Chinese medicine principles. Recently, the carbonized nanoarchitectures (CNAs) were discovered in carbonized drugs [12]. These CNAs are spherical carbon nanomaterials with a size of less than 10 nm, whose structure is similar to that of nanobiochar particles or carbon-based quantum dots [13, 79]-align with our core argument that self-assembled nanostructures represent a crucial, yet underexplored, material basis of TCM efficacy. Except for hemostatic effects, CNAs exhibited multiple therapeutic effects, including anti-inflammatory, anti-gout, hypoglycemic, liver protection, analgesic, antiviral, anti-frostbite, and antibacterial activities. Notably, while these diverse activities are promising, the specific structural features of CNAs (*e.g*., surface functional groups, carbon core composition) that mediate each effect remain poorly defined, limiting our ability to fully link their nano-scale properties to TCM’s traditional therapeutic value.

### 4.1 CNAs Exerting Hemostatic Effect

Typhae Pollen, derived from the dried pollen grains of *Typha angustifolia* L., *Typha orientalis* Presl, or related species within the same genus, has been traditionally utilized in Asian folk medicine. Its carbonized form, Pollen Typhae Carbonisata (PTC), officially listed in the *Chinese Pharmacopoeia* (2020 edition), serves as a clinically recognized hemostatic agent. Yan *et al* [80] discovered CNAs from aqueous extracts of PTC and assessed their anti-hemorrhagic effects. PTC-CNAs (2-8 nm in size) showed a remarkable anti-hemorrhagic effect in the mouse tail amputation and liver scratch models. Additionally, PTC-CNAs could increase the fibrinogen and the platelet count and then decrease the activated partial thromboplastin time. To standardize the quality of PTC-CNAs, carbonization parameters were systematically investigated [81, 82]. The optimal heating temperature and processing time were 272.35 and 7 min, respectively. Thermal treatment progressively reduced glycoside levels (typhaneoside and isorhamnetin-3-O-neohespeidoside) while elevating aglycone concentrations (isorhamnetin). Notably, two novel compounds (huaicarbon A/B) reached peak levels after 15 minutes of heating and then declined by the 35-minute mark. Simultaneously, it was necessary to control the quality of PTC-CNAs to explore the relationship between the carbonized process and its anti-hemorrhagic effects [83].

Cirsii Herba and Cirsii Japonici Herba are two herbs of *Cirsium* genus, which are traditionally used for cooling blood and hemostasis. Qu’ group [84, 85] discovered and purified CNAs from aqueous extracts of Cirsii Herba Carbonisata and Cirsii Japonici Herba Carbonisata, respectively. The CNAs derived from these herbs displayed quasi-spherical morphology (1.0~5.0 nm) with low RAW 264.7 cell toxicity. Hemostasis assays involving caudal resection and hepatic laceration revealed markedly reduced bleeding durations versus controls. The haemostatic mechanism may involve stimulation of both fibrinogen conversion and the tissue factor-dependent coagulation pathway. Junci Medulla Carbonisata (JMC), derived from the carbonized pith of *Juncus effusus* L. stems, is listed in the Chinese Pharmacopoeia (2020 edition) for its ability to clear heart fire, thereby alleviating insomnia, oliguria, and oral ulcers. Cheng *et al* [86] prepared JMC-CNAs to explore their hemostatic and hepatoprotective bioactivity. JMC-CNAs were reported to possesse remarkable hemostatic effect in mice internal hemorrhage model and trauma hemorrhage model. JMC-CNAs additionally lowered hepatic enzyme levels (ALP, ALT, AST) and bilirubin concentrations (both direct and total), demonstrating protective effects against hemorrhage-related liver damage.

Schizonepetae Herba Carbonisata (SHC) and Schizonepetae Spica Carbonisata (SSC) are carbonized aerial parts and carbonized spica of *Schizonepeta tenuifolia* Briq., respectively. SHC and SSC have served as hemostatic agents for centuries and were officially recorded in the 2020 edition of the Chinese Pharmacopoeia. Moreover, modern pharmacological studies showed they could treat various hemorrhage syndromes, such as haematochezia, postpartum haemorrhage, and metrorrhagia [87]. SHC-CNAs and SSC-CNAs were synthesized through an optimized pyrolysis approach, exhibiting particle sizes ranging from 1 to 10 nm with quantum yields between 2% and 6% [88, 89]. Mice tail haemorrhaging and liver haemorrhaging experiments showed they had significantly shorter bleeding time *via* platelet elevation. These NAs exhibited similar structural and physicochemical properties, such as diameter, fluorescence, and water solubility. Carbonisata Selaginellae Herba (CSH), the carbonized herb of *Selaginella tamariscina*, demonstrates clinically validated hemorheological enhancement properties in traditional Chinese therapeutics. The carbonized substance of CSH had been confirmed to exert a hemostatic effect [90]. CSH-derived CNAs exhibited a size range of 1.4-2.8 nm with minimal cytotoxicity toward RAW 264.7 macrophages [91]. Their hemostatic activity involved enhancement of both the extrinsic coagulation cascade and fibrin formation, along with elevated fibrinogen levels and platelet counts. CNAs from Phellodendri Chinensis Cortex Carbonisata showed a similar hemostatic effect [92]. Zhang *et al* [93] found that Scutellariae radix carbonisata-derived CNAs exhibited therapeutic effects in rat models of hematologic disorders and acute bleeding. CNAs derived from carbonized herbs possess the same physical structure as carbon quantum dots, and their hemostatic effect is associated with flavonoids [94, 95]. CNAs established an innovative approach to investigate the bioactive constituents of carbonized herbal hemostatics, while creating translational potential for managing clinical bleeding disorders.

### 4.2 CNAs with Other Bioactivities

**Fig. (4):**
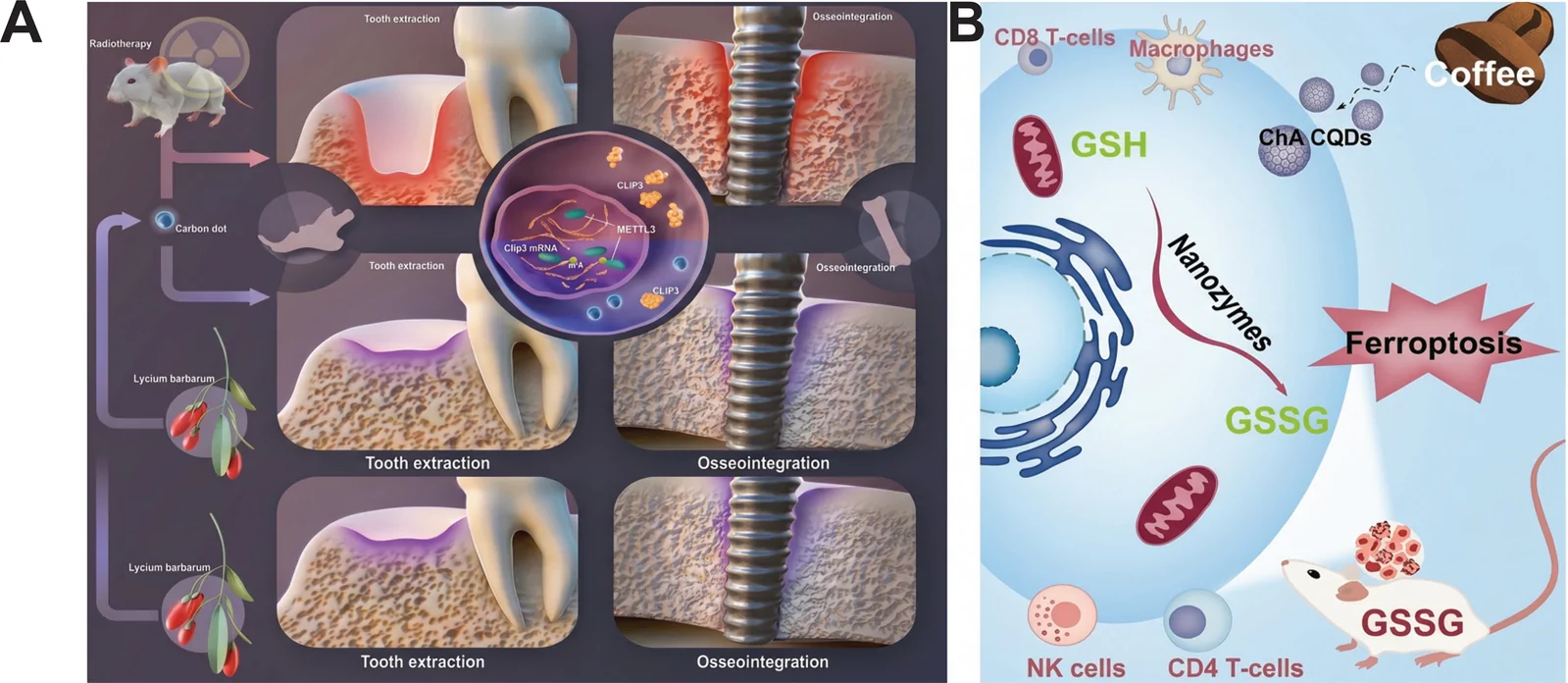
(**A**) Carbon dots from *Lycium barbarum* attenuating radiation-induced bone injury. Reproduced with permission from Ref. [118]. Copyright 2023, American Chemical Society; (**B**) Carbon dots-based nanozyme derived from coffee triggers ferroptosis and antitumor immunity. Reproduced with permission from Ref. [121]. Copyright 2022, American Chemical Society.

Although the pyrolysis approach has been widely employed to study carbonized botanicals' coagulation properties, this methodology predominantly focuses on a limited spectrum of medicinal plants and their therapeutic possibilities. Luo *et al* [96] synthesized biocompatible Taoren-Honghua carbon dots (TH-CDs) using an eco-friendly hydrothermal approach, with Semen pruni persicae and Carthamus tinctorius L. as sustainable precursors, eliminating organic solvent requirements. TH-CDs, representing a promising TBI therapeutic approach, demonstrated efficacy in enhancing neurological recovery, reducing cerebral edema, mitigating neuronal injury, and stabilizing blood-brain barrier integrity when administered intravenously in murine models, while maintaining an excellent safety profile. The expression of claudin-5 and ZO-1, key tight junction components, was enhanced by TH-CDs, possibly through their electrostatic binding with claudin-5 (Fig. **4A**). This neuroprotective effect aligns with the traditional use of *Semen pruni persicae* and *Carthamus tinctorius* L. for “promoting blood circulation” and “relieving blood stasis”—and our core argument that CNAs (or CD derivatives) are a nano-scale material basis for these effects. However, the study does not compare TH-CDs to the traditional herbal combination (Taoren-Honghua decoction) in terms of TBI efficacy, leaving a gap between modern nano-scale research and traditional TCM practice.

Crinis Carbonisatus (Xue-yu-tan), a traditional Chinese medicinal material prepared through controlled carbonization of human hair, has been empirically used for millennia in China to alleviate pain and manage neurological conditions, including epilepsy and stroke. Recent research successfully extracted and identified carbon dots (CrCi-CDs) in this substance. Zhang *et al* [97] demonstrated that CrCi-CDs exhibit neuroprotective properties against cerebral ischemia-reperfusion injury, potentially mediated through anti-inflammatory pathways. These findings indicate CrCi-CDs’ therapeutic potential as adjuncts to thrombolytic agents in acute stroke management.

Lonicerae Japonicae Flos Carbonisata (LJFC), the carbonized flower bud of *Lonicera japonica* Thunb., is used for detoxifying (defervescence and anti-inflammation) and related symptoms [98]. Wu *et al* [99] demonstrated that LJFC-CNAs effectively counteracted LPS-induced thermoregulatory disturbances in rat models, showing both antipyretic and anti-hypothermic effects. These carbon-based nanomaterials demonstrated notable anti-inflammatory effects, primarily by suppressing key inflammatory mediators (TNF-α, IL-1β, and IL-6), thereby reducing fever symptoms. CNA derived from carbonized Bombyx mori cocoons [100] exhibited inflammation-suppressing effects through IL-6 and TNF-α inhibition, with bioactivity matching that of pyrolyzed silkworm cocoons. Sophorae Flavescentis Radix Carbonisata (SFRC), the carbonized root of *Sophora flavescens* Ait., has been widely used to treat abundant ulcerative diseases [101]. SFRC-CNAs were reported to demonstrate dual anti-inflammatory and antioxidant effects in ethanol-induced gastric ulcers. They significantly suppressed pro-inflammatory mediators (NF-κB, TNF-α) and iNOS expression, while modulating oxidative markers (glutathione restoration, malondialdehyde reduction) [102]. Subsequently, CNAs were obtained from Paeoniae Radix Alba Carbonisata by Zhao’s team [103] to investigate their liver-protective properties. These CNAs with 1.0-2.4 nm in size were found to decrease the levels of alanine transaminase and aspartate aminotransferase and increase the levels of total bile acids and total bilirubin in the CCl_4_-triggered acute hepatic injury model. Notably, these CNAs reportedly demonstrated dual antioxidant capacity by eliminating reactive oxygen species and inhibiting lipid oxidative damage, thereby providing hepatoprotection. Puerariae Lobatae Radix Carbonisata derived CNAs (PLRC-CNAs) were evaluated for anti-gout effects using hyperuricemic and arthritic rodent models [104]. PLRC-CNAs were nearly spherical with size of 3-10 nm and were observed to reduce serum urate concentrations by inhibiting xanthine oxidoreductase activity. Moreover, PLRC-CNAs have been proposed to serve as effective nanocarriers that enhance aqueous dissolution of baicalin, potentially due to structural interactions with its glucuronyl moiety [105]. CNAs derived from Aurantii Fructus Carbonisata were also reported to exhibit urate-lowering and gout-alleviating effects [106]. These bioactive CNAs are suggested to show excellent safety profiles, supporting their translational potential for gout therapy using carbonized traditional medicines.

Jiaosanxian (JSX) is a carbonized herbal formula, including Crataegi Fructus Carbonisata, Hordei Fructus Germinatus Carbonisata, and Massa Medicata Fermentata Carbonisata, which is widely used for treating dyspepsia in China [107]. JSX derived CNAs were shown to have prominent hypoglycemic activity in hyperglycemic mouse model [108]. Interestingly, CNAs from Crataegi Fructus Carbonisata, one drug of JSX, also exhibited hypoglycemic activity [109]. These CNAs suppressed sucrase and maltase function, thereby lowering postprandial blood glucose. While this mechanism is clear, the study does not explore whether CNAs from the three JSX components act synergistically—consistent with TCM’s “Jun-Chen-Zuo-Shi” compatibility theory—or if their effects are additive. Li *et al* [110] separated CNAs from Zingiberis Rhizoma Recens that provided high selective inhibition of human liver cancer cells (HepG2). The CNAs enhanced p53 protein levels and stimulated intracellular ROS generation, triggering HepG2 cell apoptosis. In addition, these CNAs were also proposed to possess a remarkable analgesic effect, potentially involving opioid receptor modulation and 5-HT level changes [111]. Artemisiae Argyi Folium Carbonisata (AAFC) is derived from carbonized leaf of *Artemisia argyi* Levl. Et Vant., which has been used to prevent plague, inflammatory diseases, and tumor. AAFC-derived CNAs improved cold tolerance in mice by inhibiting pro-inflammatory cytokines and stabilizing glucose levels post-frostbite [112]. Additionally, AAFC-CNAs demonstrated targeted antimicrobial activity against specific Gram-negative pathogens, including E. coli, P. aeruginosa, and P. vulgaris [113]. AAFC-CNAs suppressed key enzymatic functions essential for bacterial cell wall integrity, leading to structural damage. However, the study does not assess whether AAFC-CNAs affect beneficial commensal bacteria (*e.g*., gut lactobacilli), raising concerns about potential disruption of microbial homeostasis. CNAs derived from Forsythiae Fructus showed anti-wood rot fungus activity [114]. Antimycotic efficacy toward *G. trabeum* and *C. versicolor* reportedly matched conventional ammoniacal copper quats preservative performance. Liu *et al* [115] demonstrated that GR-derived CNAs significantly alleviated ethanol-induced gastric lesions in murine models by attenuating alcohol-mediated mucosal oxidative injury. Glycyrrhizic acid, the active ingredient in GR, could also become biocompatible CNAs by a hydrothermal method [116]. Glycyrrhizic acid-based CNAs were observed to significantly inhibit PRRSV replication. This antiviral action is suggested to involve modulation of DDX53/NOS3 pathways, enhanced host defense activation, and reduced oxidative stress. However, it is worth noting that being similar to glycyrrhizic acid, curcumin-based CNAs showed minimal anti-EV71 efficacy [117]. Guo *et al* [118] found that *Lycium barbarum* derived CNAs could inhibit senescence through regulating Clip3 m6A methylation, mitigating radiation-associated osseous injury (Fig. **4B**). In addition, CNAs derived from TCMs were also proposed to have anti-anxiety and certain sedative effects and could ameliorate imiquimod-induced psoriasis-like inflammation [119, 120]. Therefore, CNAs from Chinese carbonized drugs may demonstrate potential for expanding TCM’s therapeutic scope.

### 4.3 Food-derived Carbon Quantum Dots

Beyond specifically defined Chinese carbonized drugs, the concept of deriving bioactive carbon nanomaterials can be extended to common food sources. Food-derived carbon quantum dots (CQDs) are proposed to show great promise as bioactive nanomaterials with various therapeutic applications. Zeng’s group [121] synthesized coffee-originated CQD nanozymes using CGA, which exhibited glutathione-oxidizing capacity through GSH-to-GSSG conversion. This redox modulation triggered ferroptotic cell death by depleting glutathione reserves and suppressing GPX4 activity. *In vivo*, CGA CQDs greatly inhibited tumor growth *via* noticeably enhancing ferroptosis-dependent immune cells’ infiltration to turn immunologically “cold” tumors into “hot” tumors (Fig. **4B**). Wen *et al* [122] successfully synthesized and analyzed green tea-derived CQD-curcumin complexes, reportedly demonstrating enhanced curcumin antioxidant capacity while preserving its antimicrobial efficacy. This suggests curcumin-loaded tea CQDs could serve as dual-functional natural agents with both antioxidative and antibacterial properties. Such discoveries underscore the multifunctional applications of dietary-source carbon nanomaterials in developing novel therapeutic systems combining antioxidant, antimicrobial, and anticancer effects, although their pharmacokinetics and long-term safety are not yet fully established.

### 4.4 Critical Challenges and Future Perspectives

The research on CNAs faces unique hurdles. A primary issue is the ambiguous structure-activity relationship. While often called “carbon dots”, their precise molecular structure, surface functional groups, and the role of inherent inorganic ions are poorly defined. Are the bioactivities intrinsic to the carbon core, or conferred by surface residues from the precursor herbs? Secondly, the pharmacokinetics and long-term safety profile of these carbon-based nanomaterials are virtually unknown. Their *in vivo* fate, biodistribution, and potential for accumulation require thorough investigation before any clinical translation can be contemplated. Future work must focus on correlating specific carbonization parameters (temperature, atmosphere, duration) with precise structural features and biological outcomes to move from phenomenological discovery to controllable design.

## 5 SELF-ASSEMBLED EXTRACELLULAR VESICLE-LIKE NANOPARTICLES FROM CHINESE FRESH HERBS

Fresh plant-derived extracellular vesicle-like nanoparticles (EVNs) are nanoscale structures ranging from 40 to 150 nm, produced by plant cells. These vesicles carry cellular components such as proteins, lipids, and nucleic acids, functioning as key mediators in cell-to-cell communication to maintain plant physiological processes [14, 15, 123]. Given their advantage of large-scale producibility, EVNs derived from Chinese fresh herbs are attracting increasing research attention for disease therapy, though this interest remains largely driven by preliminary *in vitro* and small-animal studies, with clinical translation still in its infancy.

### 5.1 EVNs Derived from Zingiberis Rhizoma Recens

Zingiberis Rhizoma Recens (ZRR), the fresh rhizome of *Zingiber officinale* Rosc., has been traditionally used in Chinese medicine to induce sweating for cold relief and warm the lungs to alleviate coughing. Research [124] indicates that ZRR is effective against nausea and gastrointestinal disorders, including colic, bloating, and loose stools. ZRR-derived EVNs exhibited a mean particle diameter of 230 nm with a negatively charged surface, carrying components such as lipids, proteins, microRNAs, and active constituents like 6-gingerol and 6-shogaol [125]. ZRR-EVNs were shown to alleviate severe intestinal inflammation and promote gut tissue regeneration in murine models of colitis. When administered orally, ZRR-EVNs stimulated growth of gut lining cells by elevating levels of IL-10 and IL-22 while suppressing TNF-α, IL-6 and IL-1β. Intranasal delivery of these nanoparticles showed protective effects against lung scarring caused by bleomycin in mice, demonstrating free radical-scavenging, inflammation-modulating and protein-digesting properties [126]. Notably, the long-term pulmonary safety of intranasal ZRR-EVNs (*e.g*., potential for chronic inflammation or tissue accumulation) was not evaluated, which is critical for their use in treating chronic lung conditions. Yin *et al* [127] found 27 significantly upregulated miRNAs in ZRR-EVNs. These nanoparticles demonstrated anti-inflammatory effects against LPS stimulation through suppression of NF-κβ signaling and reduced production of inflammatory mediators, including IL-6, IL-8, and TNF-α. These protective effects are proposed to be attributed to the abundant miRNA content within the vesicles. Next, ZRR-EVNs could ameliorate mouse colitis by shaping the gut microbiota [128]. ZRR-EVNs were first taken up by *Lactobacillus rhamnosus*. The miRNAs present in ZRR-EVNs were found to specifically bind monooxygenase ycnE, elevating levels of indole-3-carbaldehyde. This subsequently enhanced IL-22 generation, thereby promoting anti-inflammatory effects.

Chen *et al* [129] revealed that the bioactive lipids in ZRR-EVNs (excluding nucleic acid/protein components) effectively blocked NLRP3 inflammasome formation. This treatment also mitigated downstream inflammatory responses mediated by NLRP3, particularly the proteolytic activation of caspase-1 and subsequent cytokine (IL-1β/IL-18) release. Beyond anti-inflammatory actions, ZRR-EVNs alleviated ethanol-induced hepatic injury in mice [130]. ZRR-EVNs mediated activation of Nrf2 to contribute to liver protection. Furthermore, 6-shogaol in ZRR-EVNs was identified to activate Nrf2 *via* the TLR4/TRIF signaling axis. This discovery revealed novel applications for ZRR in preventing hepatic disorders, particularly those caused by alcohol consumption. Additionally, ZRR-EVNs have been proposed as serve as promising candidates for managing persistent periodontal inflammation [131].

ZRR-EVNs were internalized by *Porphyromonas gingivalis*, a key periodontal pathogen, through phosphatidic acid-mediated binding to the bacterial surface protein HBP35. This binding event markedly attenuated the virulence of *P. gingivalis*. However, the study did not assess whether ZRR-EVNs affect other commensal bacteria in the oral cavity, raising concerns about potential disruption of oral microbial homeostasis. Finally, ZRR-EVNs could serve as delivery nanovehicles to load therapeutic agents to enhance their bioactivity. Chemotherapeutic drug doxorubicin was successfully loaded in ZRR-EVNs [132]. ZRR-EVNs were reported to have immensely improved the tumor targeting of doxorubicin and promoted the apoptosis of tumor cells. Besides, ZRR-EVNs were loaded with siRNA-CD98 to treat ulcerative colitis [133, 134]. These ZRR-EVNs were specifically targeted to colon tissues to reduce the expression of CD98. Thus, ZRR-EVNs are suggested to have great promise as efficient therapeutic agents-delivery vehicles to enhance their relevant bioactivity, though their stability and large-scale production remain challenging.

### 5.2 EVNs Derived from *Citrus*-herbs

**Fig. (5):**
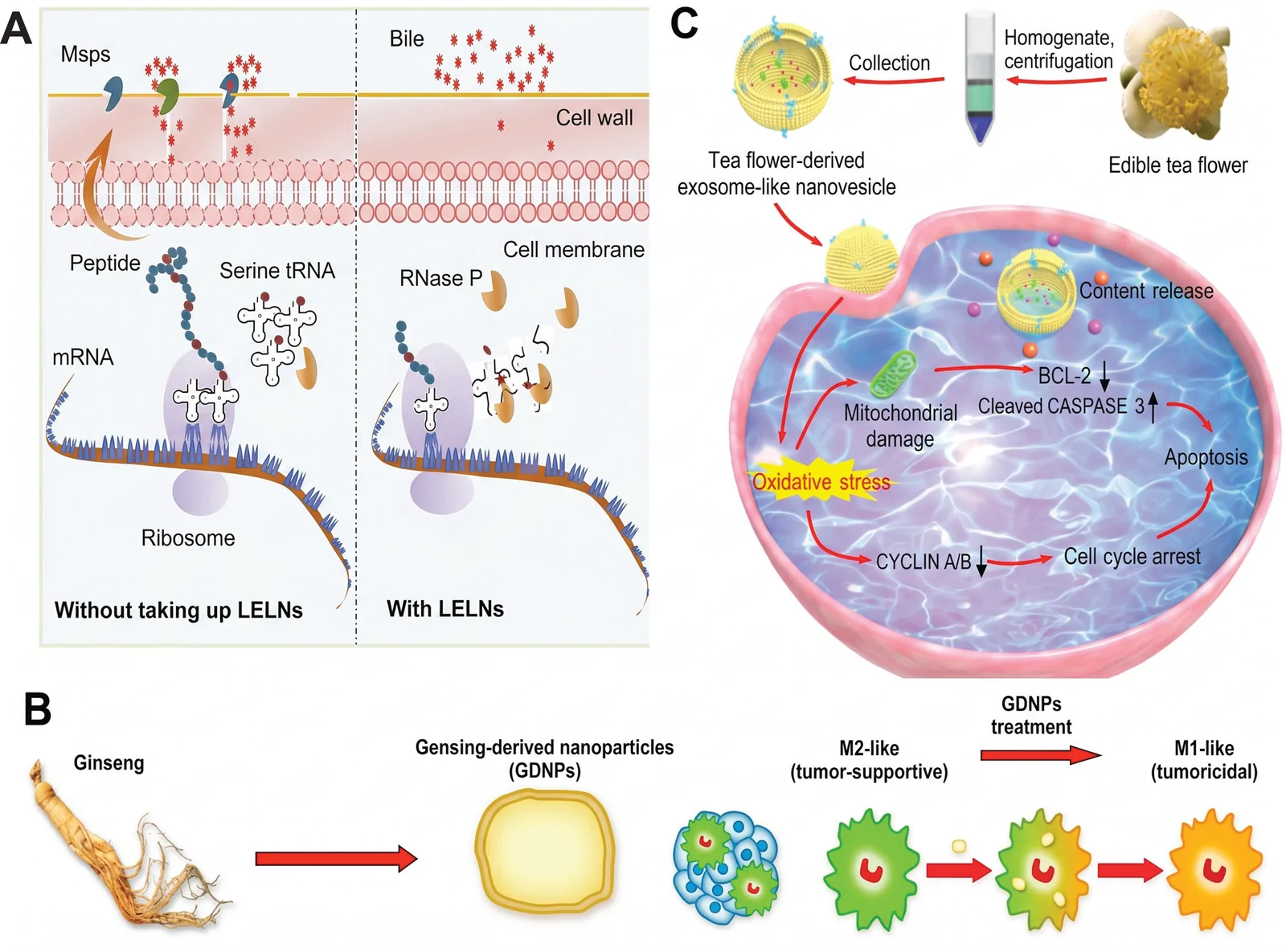
(**A**) Self-assembled extracellular vesicles from fresh *Citrus limon* improve gut microbiota resilience through RNase P-dependent tRNA cleavage. Reproduced with permission from Ref. [138]. Copyright 2021, Elsevier Inc; (**B**) Self-assembled extracellular vesicles from fresh *Panax ginseng* modulate macrophage phenotypes, suppressing melanoma progression. Reproduced with permission from Ref. [149]. Copyright 2019, Springer Nature Limited; (**C**) Self-assembled extracellular vesicles from fresh *Camellia sinensis* inhibit breast cancer metastasis through reactive oxygen species induction and gut flora regulation. Reproduced with permission from Ref. [162]. Copyright 2022, Elsevier Inc.

*Citrus*-based TCMs, such as Citri Reticulatae Pericarpium (the pericarp of *Citrus reticulata* Blanco), Aurantii Fructus Immaturus (the fruit of *Citrus aurantium* L.), Citri Sarcodactylis Fructus (the fruit of *Citrus medica* L.), and Citri Grandis Exocarpium (the pericarp of *Citrus grandis* L. Osbeck), are widely used in China to regulate the flow of Qi and resolve phlegm to relive cough. Xiao *et al* [135] successfully separated EVNs with nanometer scale from fresh fruits of *C. reticulate* using differential centrifugation. Bioinformatic analysis suggests MIR-398b in these nanoparticles could influence human mRNA regulation, resulting in anti-inflammatory activity. Next, the protein cargos in EVNs from the fruits of *C. aurantium* were analyzed using label-free quantitative shotgun proteomics [136]. These EVNs were analyzed for their content of patellin-3-like protein, clathrin heavy chain, heat shock proteins, 14-3-3 protein, GAPDH, and fructose-bisphosphate aldolase 6. Baldini *et al* [137] successfully extracted EVNs from *Citrus limon* L. (CL) juice, demonstrating particle diameters between 30 and 100 nm. CL-EVNs contained citrate, vitamin C and specific miRNAs that were internalized by mesenchymal stromal cells, demonstrating antioxidative properties. CL-EVNs could also enhance lactobacilli tolerance to bile to regulate gut microbiota [138]. CL-EVNs suppressed Msp1 and Msp3 expression by degrading tRNAserUCC and tRNAserUCG, limiting bile access to membranes. The active component was identified as pectin-type polysaccharides enriched in galacturonic acid (Fig. **5A**). Zhang *et al*. first demonstrated that CL-EVNs could inhibit kidney stone progression by alleviating endoplasmic reticulum stress in renal tubular cells [139]. Subsequent studies further revealed their broad anticancer properties [140, 141]. Notably, these nanoparticles triggered cell cycle blockage at the S-phase and stimulated caspase activation *via* ROS generation. Preclinical evaluations confirmed their potent growth-inhibitory effects on multiple cancer cell types (A549, LAMA84, SW480) through TRAIL-dependent apoptosis. Stanly *et al* [142] found nano-sized EVNs from fruit juice of *C. paradise* (CP). CP-EVNs were enriched with α-hydroxy acids, branched-chain amino acids (leucine/isoleucine), myo-inositol, and doconexent. These components demonstrated anti-melanoma effects in A375 cells by suppressing cyclin B1/B2 while enhancing p21 expression. The inherent biocompatibility and biodegradability of CP-EVNs prompted CP-EVNs as a delivery system to load methotrexate [143]. CP-EVNs enhanced methotrexate efficacy against DSS-colitis in mice by suppressing IL-1β/TNF-α production in gut macrophages while upregulating HO-1. These nanoparticles could encapsulate either chemotherapeutic doxorubicin or anti-inflammatory curcumin, both alleviating DSS-colitis inflammation [144]. While CP-EVNs show promise as a multi-drug delivery platform, their stability in gastrointestinal fluids (*e.g*., gastric acid, bile salts) was not evaluated, which is critical for maintaining drug bioavailability after oral administration. Additionally, CP-EVNs demonstrated delivery capacity for nucleic acids (siRNA, plasmid DNA) and proteins across various cell types, enhancing antitumor efficacy [145-147]. Biostability and bioavailability of the loaded siRNA and proteins were distinctly improved. CP-EVNs demonstrated lower toxicity compared to synthetic nanoplatforms, indicating their potential as effective carriers for therapeutic siRNA.

### 5.3 EVNs Derived from Other Herbs

Ginseng Radix et Rhizoma, the dried root of *Panax ginseng* (PG), exhibits diverse therapeutic benefits such as immune regulation, anticancer potential, oxidative stress reduction, inflammation control, and senescence delay [148]. Cao’ group [149, 150] isolated EVNs with abundant proteins, lipids and nucleic acids from fresh roots of PG. PG-EVNs demonstrated macrophage-mediated immunoregulation capable of suppressing melanoma progression. Notably, PG-EVNs prompted polarization of tumoricidal M1 phenotype macrophage *via* TLR4/MyD88-dependent signaling, subsequently triggering reactive oxygen species (ROS)-mediated apoptosis in melanoma cells (Fig. **5B**). They further found PG-EVNs could switch a cold tumor microenvironment to a hot tumor microenvironment to improve potent immune checkpoint inhibitor therapy. PG-EVNs modulated tumor-associated macrophage polarization, elevating CCL5/CXCL9 secretion to enhance CD8^+^ T cell tumor infiltration. These effects suggested a combinatorial strategy to treat tumors with PD-1 mAb.

Asparagi Radix, derived from *Asparagus cochinchinensis*, exhibits diverse therapeutic effects encompassing anticancer, antifungal, antioxidation, and antiaging activities [151]. Zhang *et al* [152] isolated EVNs from fresh *A. cochinchinensis* and assessed their antitumor against hepatoma carcinoma cell. Experimental studies demonstrated these EVNs suppressed tumor cell growth, induced programmed cell death, and enhanced expression of apoptotic mediators while showing minimal cytotoxicity toward normal cells. EVNs derived from *Dendropanax morbifera* showed strong cytotoxic effects on tumor cells (A431, MCF-7 and MDA-MB-231 cells) [153]. Concentration-dependent suppression of melanin synthesis and tyrosinase function was achieved through downregulation of MITF, TRP-1, and TRP-2 expression [154], suggesting their potential as innovative skin-lightening agents, though clinical efficacy remains to be proven.

Taraxacum officinale (T. officinale), a medicinal and edible homologous plant, demonstrates potent inflammation suppression and oxidative stress mitigation capabilities, with therapeutic potential for managing persistent illnesses and immune dysfunction. Its exosomes ameliorated gut inflammation and protected intestinal integrity through microbiome modulation and short-chain fatty acid regulation, subsequently preventing systemic inflammatory responses and vascular remodeling in hypoxia-associated hypertension [155].

Mulberry leaves possess therapeutic benefits, including oxidative stress reduction, blood sugar control, microbial inhibition, and inflammation suppression. Researchers isolated biologically derived lipid nanoparticles from *Morus nigra L.* (black mulberry) leaves, showing uniform nanoscale dimensions (162.1 nm diameter, PDI 0.025) and negative surface charge (-26.6 mV). These plant-based nanoparticles, when administered orally, effectively treated liver cancer through oral administration by elevating cellular ROS and disrupting mitochondrial function [156].

**Fig. (6):**
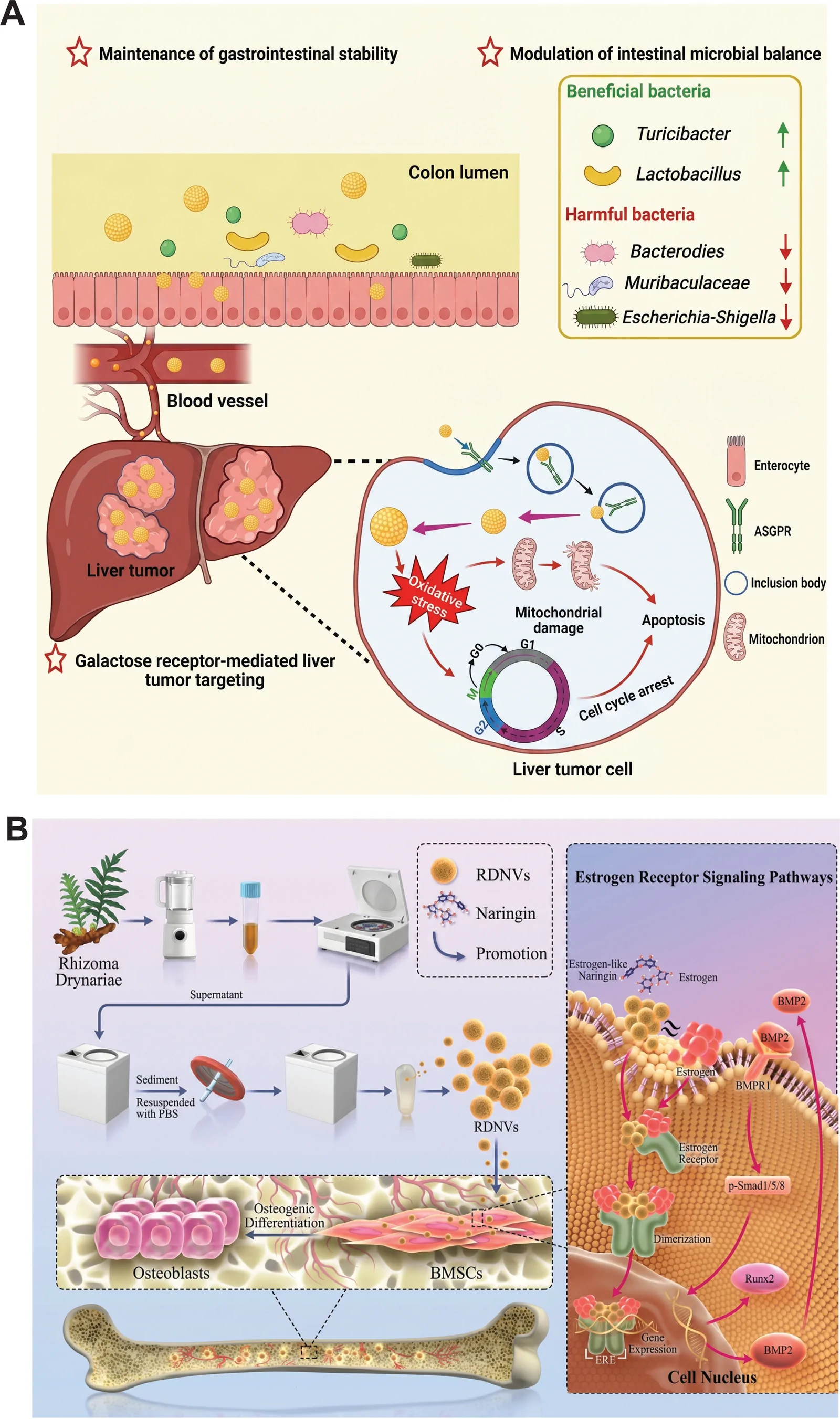
(**A**) Natural lipid nanoparticles extracted from *Morus nigra* L. leaves for targeted treatment of hepatocellular carcinoma. Reproduced with permission from Ref. [156]. Copyright 2024, Springer Nature; (**B**) Nanovesicles derived from *Rhizoma Drynariae* alleviate bone loss by stimulating osteogenesis in bone marrow mesenchymal stem cells *via* estrogen receptor signaling. Reproduced with permission from Ref. [158]. Copyright 2024, Elsevier Inc.

Brucea javanica (L.) Merr., a renowned medicinal plant, has demonstrated potent anticancer activity against a variety of cancer cell types. Current research from Yan’s group [157] successfully purified extracellular vesicles from Brucea javanica (termed BF-Exos), investigating their miRNA delivery capabilities. These BF-Exos have shown promise as a natural, biocompatible nanoplatform that not only facilitates miRNA delivery but also plays a dual role in combating TNBC by synergistically suppressing both angiogenesis and tumor progression (Fig. **6A**).

Rhizoma Drynariae, a medicinal herb, combines analgesic and osteogenic effects, offering therapeutic benefits for osteoporosis and fracture repair. Zhao *et al* [158] demonstrated the medicinal value of nanovesicles from Rhizoma Drynariae (RDNVs) for osteoporosis in postmenopausal women. Their study found these vesicles promote bone-forming differentiation in hBMSCs by modulating estrogen receptor (ERα). Further analysis identified naringin, a key component in RDNVs, as the active compound mediating this osteogenic effect specifically through targeting ERα (Fig. **6B**).

Pueraria lobata, a traditional herb, exhibits therapeutic potential for central nervous system disorders. The active compound puerarin helps maintain mitochondrial integrity by preserving membrane polarization, decreasing oxidative stress, and restoring defective autophagy of damaged mitochondria. Notably, this traditional therapeutic potential is now being linked to the herb’s self-assembled nanostructures: exosomes isolated from P. lobata (Pu-Exos) act as effective nanocarriers for transporting bioactive macromolecules into Parkinson’s disease model SH-SY5Y cells, aligning with our core argument that self-assembled nanostructures are a crucial material basis of TCM efficacy. These vesicles markedly ameliorate neuronal metabolic deficits in dopaminergic cells by delivering regulatory factors that enhance PINK1/Parkin-dependent mitochondrial quality control and boost electron transport chain components I and V, suggesting promising neuroprotective applications [159].

Goji berry (*Lycium barbarum* L.), known as “Gou qi zi”, is a medicinal fruit documented in ancient Chinese pharmacopoeia for its longevity-promoting effects and capacity to improve musculoskeletal integrity. Consistent with our overarching thesis, modern research has uncovered that this traditional efficacy may stem from self-assembled nanostructures: Zhou *et al* [160] isolated GqDNVs from fresh Goji berries and demonstrated their ability to enhance skeletal muscle performance through modulation of the AMPK-dependent metabolic signaling cascade. This suggests that GqDNVs may serve as a potential anti-aging agent for skeletal muscle, offering a promising approach for future therapy and research on muscle aging. However, the study does not directly compare GqDNVs to other components of Goji berries (*e.g*., polysaccharides or flavonoids) in terms of muscle-protective efficacy, leaving uncertainty about the unique contribution of these nanostructures to the fruit’s traditional effects.

Artemisia annua, known for its anti-malarial effects, also shows immunoregulatory and anti-tumor properties. Shi’s group [161] identified extracellular vesicles from artemisia, naming them ADNVs (artemisia-derived nanovesicles). These nanoparticles demonstrated remarkable antitumor efficacy in lung cancer models, exhibiting both cytostatic effects and immunomodulation activity. ADNVs modulate immune cells in the tumor microenvironment through phytogenic organelle genetic material, triggering the cyclic GMP-AMP synthase signaling axis. Furthermore, ADNVs improved the efficacy of PD-L1 inhibitors, suggesting their potential in cancer. This finding reinforces our core argument: self-assembled nanostructures like ADNVs are not just byproducts of TCM, but likely key mediators of its therapeutic effects—bridging the gap between artemisia’s traditional use and modern cancer immunotherapy.

### 5.4 EVNs Derived from Medicinal and Edible Plants

Building on the link between self-assembled nanostructures and TCM efficacy established in the preceding sections, the following section focuses on EVNs from medicinal and edible plants-a category that embodies TCM’s “food as medicine” philosophy, where self-assembled nanostructures may similarly underpin the health-promoting effects of these dual-purpose materials.

Xinnaojian tablet or capsule, recorded in the 2020 Pharmacopoeia of China, has been used for the treatment of dizziness, chest shortness of breath, fatigue, lethargy, mental loss, and memory loss, of which main components are extracts of *Camellia sinensis* (Linn.) O. Ktze. In line with our thesis that self-assembled nanostructures are a critical material basis of TCM efficacy, Chen *et al*. [162] successfully extracted extracellular vesicles (EVs) from *Camellia sinensis* floral sources, demonstrating selective pro-apoptotic effects to breast tumors. These EVNs induced oxidative stress buildup, causing impaired energy metabolism and consequent proliferation arrest (Fig. **5C**).

EVNs were separated from juice of *Vitis vinifera* L., containing proteins, lipids, and microRNA [163, 164]. Subsequent cellular uptake experiments demonstrated their capacity to selectively accumulate in intestinal macrophages, triggering TCF4-mediated activation of canonical Wnt signaling. Furthermore, *V. vinifera*-derived EVNs exerted protective effects against DSS-triggered colonic inflammation by targeting intestinal stem cells [165]. Lipid components of EVNs promoted the expansion of intestinal progenitor cells *via* canonical Wnt signaling and were essential for their *in vivo* tropism toward the stem cell niche. This anti-inflammatory effect aligns with the traditional use of grapes (and grape-derived products) in TCM to “nourish the blood” and “soothe the intestines”, and our core argument that self-assembled nanostructures like EVNs are key to explaining such effects at the nano-scale. *Heliantus annuus* L. could also secrete functional EVNs carrying the GTPase Rab protein, of which receptacle, root, stem pith, leaf and seed were generally used to reduce blood pressure, relieving pain, and relieving cough and asthma [166]. Zhao *et al* [167] isolated EVNs from *Cocos nucifera* L. and demonstrated the existence of microRNAs in them. These extracellular vesicles (EVs) may be internalized by animal cells, subsequently modulating their proliferation and metabolic activities. EVNs derived from *Lentinula edodes* alleviated hepatotoxicity in a chemically induced hepatic damage model by suppressing NLRP3 inflammasome activation [168]. Shiitake is widely used in TCM to “invigorate Qi” and “protect the liver”, and this study provides evidence that EVNs may be part of the material basis for this liver protection—consistent with our argument that self-assembled nanostructures are underexplored mediators of TCM efficacy. Fragaria ananassa-derived EVNs were enriched with anthocyanin compounds, folate derivatives, flavonol glycosides, and ascorbic acid, demonstrating dose-responsive antioxidant effects indicative of potential health-promoting activity [169]. Deng *et al* [170] prepared broccoli-derived EVNs and affirmed their efficacy in regulating intestinal immune homeostasis. Broccoli EVNs modulated AMPK activity in DCs, offering protection against DSS-mediated colonic inflammation. *Cucumis sativus*-derived EVNs could enhance transdermal delivery of hydrophobic compounds [171], while *Brassica oleracea*-derived EVNs could effectively deliver anti-inflammatory agents to immunocytes and induce programmed cell death in skin cells [172]. Garlic-derived EVNs can stimulate natural proliferation and functional enhancement of γδT lymphocytes following oral delivery. This modulates the tumor immunological milieu and potentiates PD-L1 blockade efficacy, generating robust tumoricidal immunity [173]. Collectively, these findings demonstrate EVNs' promise as innovative bioactive therapeutics—and more importantly, they reinforce our core argument that self-assembled nanostructures represent a crucial, yet underexplored, material basis of TCM efficacy, providing a nano-scale perspective that bridges traditional philosophy (*e.g*., “food as medicine”) and modern science. Though their heterogeneity and isolation reproducibility pose significant challenges, these issues are not unique to medicinal and edible plant EVNs but rather reflect broader gaps in the field that must be addressed to fully translate TCM’s nanostructural potential.

### 5.5 Critical Challenges and Future Perspectives

The translation of EVNs into therapeutics is exciting but fraught with challenges. A significant bottleneck is the scalable and standardized production. Current isolation methods (*e.g*., ultracentrifugation) are low-yield and impractical for large-scale manufacturing. Developing efficient and reproducible bioprocessing techniques is crucial. Secondly, stability during storage and gastrointestinal transit is a major concern. Their phospholipid bilayer is susceptible to degradation. Engineering strategies to enhance their stability without compromising bioactivity are needed. Lastly, the exact mechanisms of cellular uptake and cross-kingdom communication, while intriguing, are not fully understood. Rigorous studies are required to identify specific targeting motifs on EVN surfaces and to conclusively prove the functional delivery of their RNA cargo into mammalian cells.

## 6 DISCUSSION

While the current research landscape is largely promising and descriptive, it is imperative to critically examine the field’s limitations to guide rigorous future investigations. TCM represents a profound cultural heritage that integrates ancient health philosophies with long-standing clinical practices. For centuries, it has contributed to human health through its distinctive theoretical systems and treatment methodologies [1-3]. Although bioactive small molecules are considered central to the efficacy of TCM, their therapeutic impact is strongly influenced by their physical forms and phase states—fundamental aspects that constitute the material basis of TCM. The effectiveness of TCM arises from a dual mechanism: the multi-component interactions among constituents and the distinct morphological architectures they assemble into, both of which collectively determine therapeutic outcomes. Throughout this review, we argue that these self-assembled nanostructures represent a crucial, yet underexplored, material basis of TCM efficacy, providing a novel nano-scale perspective that bridges traditional philosophy and modern science.

Herbal extracts in TCM are heterogeneous dispersion systems, consisting of molecular solutions, colloidal dispersions, emulsions, and suspensions. A major distinction from synthetic chemical approaches is the spontaneous supramolecular self-organization of TCM constituents during decoction. This process entails the autonomous arrangement of molecular entities into nanostructures through non-covalent interactions—such as hydrogen bonding, hydrophobic effects, π–π stacking, and electrostatic forces—without external intervention (Table **1**). Such self-assembly not only improves the solubility of lipophilic compounds but also enhances synergistic interactions [24, 25, 174, 175] and reduces potential toxicity [176]. Beyond small molecules, proteins and polysaccharides also participate in the formation of nanoaggregates [177, 178]. For instance, herbal proteins—with exposed polar residues and concealed nonpolar cores—improve the solubility of poorly soluble compounds. Polysaccharides, capable of forming multiple hydrogen bonds, interact with various building blocks to generate polymorphic nanostructures.

The formulation of multi-herb remedies is not a mere combination of single herbs but a dynamic process guided by TCM compatibility principles (“Jun-Chen-Zuo-Shi”), resulting in novel supramolecular configurations. Single-herb decoctions contain self-assembled structures shaped by intrinsic biomolecular interactions. For example, CRD-NAs, which are primarily composed of negatively charged polysaccharides, facilitate the absorption of BBR by modulating intestinal tight junctions [52-54]. Similarly, GRD-NAs involve 23 distinct proteins that serve as carriers, improving the bioavailability of sparingly soluble compounds such as astragaloside IV [56]. These assemblies depend exclusively on intra-herb interactions. In contrast, multi-herb decoctions incorporate a broader set of molecular building blocks, yielding more complex and functionally enhanced nanostructures through cross-component interactions not possible in single-herb preparations. A notable example is HLJDD, where unique BBR-BA nanoparticles form *via* acid-base neutralization, hydrophobic interactions, and electrostatic attraction—structures absent in individual decoctions of Coptidis Rhizoma or Scutellariae Radix [21-24]. These nanoparticles were reported to demonstrate strengthened antibacterial action against *Staphylococcus aureus* and exhibit synergistic benefits in managing IBS-D [24, 25], although a direct comparative assessment of the nano-assembly versus the physical mixture of individual components at an equivalent dose was not fully provided.

Compatibility principles in TCM directly modulate supramolecular organization. In cases of synergistic enhancement (Xiang Xu/Xiang Shi), formulations such as BHD give rise to nanoaggregates that incorporate mangiferin (from *Anemarrhenae Rhizoma*), glycyrrhizic acid (from GR), starch (from japonica rice), and Ca^2+^/Si^2+^ ions (from gypsum). Here, starch forms a colloidal framework, ions help stabilize the nano-phase, and glycyrrhizic acid functions as a natural surfactant—together augmenting antipyretic effects through suppression of IL-1β and TNF-α [29, 30]. For detoxification (Xiang Sha/Xiang Wei), as observed in Glycyrrhiza Radix–Aconiti Radix combinations, glycyrrhiza proteins engage with toxic aconitine *via* electrostatic and hydrophobic associations, forming aggregates that mitigate acute toxicity through controlled release [62]. Conversely, incompatible herb pairs may produce insoluble aggregates that diminish the bioavailability of active components; adverse interactions in poorly matched formulations can lead to precipitation of crucial compounds, thereby negating therapeutic benefits.

Carbonized TCM materials, produced through high-temperature processing, constitute a unique category of herbal medicine with a two-millennia history of use in hemorrhagic conditions [78]. Their efficacy is largely due to carbonized nanoarchitectures (CNAs)—spherical carbon nanomaterials under 10 nm in size that resemble carbon quantum dots [12, 13, 79]. The therapeutic applicability of CNAs extends beyond hemostasis. For instance, Lycium barbarum CNAs ameliorate radiation-induced bone damage by modulating Clip3 m^6^A methylation [118], while PLRC-CNAs lower serum urate levels *via* inhibition of xanthine oxidoreductase, offering anti-gout benefits [104]. Additionally, GR-CNAs guard against ethanol-induced gastric lesions through anti-inflammatory and antioxidant activities [115]. These results affirm carbonization as a viable means of producing bioactive nanostructures with diminished toxicity.

EVNs isolated from fresh herbs are gaining attention as natural therapeutic agents and drug delivery vehicles [14-16, 123]. These EVNs encapsulate lipids, proteins, miRNAs, and metabolites, facilitating intercellular communication and displaying tissue-specific targeting. ZRR-EVNs ameliorate colitis by inducing IL-10/IL-22 secretion, suppressing TNF-α/IL-1β, and modulating gut microbiota through miRNA-guided regulation of *Lactobacillus rhamnosus* [125, 128]. They also mitigate ethanol-induced liver damage by activating Nrf2 *via* TLR4/TRIF signaling [130]. CL-EVNs bolster gut microbiota resilience through RNase P-mediated tRNA cleavage, countering bile acid-induced membrane disruption [138], and curtail kidney stone development by reducing endoplasmic reticulum stress [139]. PG-EVNs shift macrophage polarization toward tumoricidal M1 phenotypes *via* TLR4/MyD88 pathways, inducing ROS-dependent apoptosis in melanoma and augmenting the efficacy of immune checkpoint inhibitors [149, 150]. These instances underscore the potential of EVNs as naturally derived nanocarriers that exploit innate biocompatibility and targeting specificity; however, limitations remain regarding their batch-to-batch consistency and long-term stability.

TCM processing (Paozhi) profoundly influences nanostructural properties, consistent with the axiom that “different processing methods yield distinct clinical outcomes.” Fresh herbs conserve intact EVNs with native lipid bilayers and complete molecular cargo (RNAs, proteins), conferring high biological activity but constrained stability; their lipid coatings shield contents from gastrointestinal degradation and promote endocytic absorption [125, 137]. Boiling during decoction disrupts most EVNs, yet the resulting fragments reorganize with polysaccharides and tannins into stable “supramolecular assemblies.” For example, HLJDD-NAs and BHD-NAs, formed through multi-component interactions, display improved stability and mucoadhesion, extending their retention in the gut [21, 27]. High-temperature carbonization, conversely, eliminates EVNs, producing microcharcoal particles with adsorbed compounds whose actions depend mainly on adsorption (*e.g*., toxin clearance) and localized hemostasis, with little systemic targeting [80, 92].

Notwithstanding these advances, significant challenges persist. Current research on TCM supramolecular assemblies remains at an early stage, and advanced methodologies—such as cryo-electron microscopy and single-molecule imaging—are required to elucidate their structural features and assembly mechanisms. Environmental variables (*e.g*., temperature, pH) influence assembly stability, and protocols for storage and large-scale manufacturing require refinement. The relationship between supramolecular structures and traditional indications (*e.g*., “heat-clearing” or “Qi-invigorating”) warrants further exploration. For EVNs from fresh herbs, stability must be assessed across seasonal variations, and scalable cultivation methods such as hydroponics or fermentation systems should be developed. Tackling these issues will improve quality control in TCM and promote the clinical translation of supramolecular assemblies.

## CONCLUSION

In conclusion, bioactively self-assembled nanoplatforms derived from TCM-including nanoaggregates, CNAs, and EVNs-provide a new perspective for understanding the material foundation of TCM. As mechanistic insights continue to accumulate, these nanostructures offer considerable potential to modernize TCM, integrating historical wisdom with contemporary nanomedical science.

## Figures and Tables

**Table 1:** Self-assembled supramolecule nanoplatforms existed in traditional Chinese medicine.

**Nanoplatform**	**Source**	**Size (nm)**	**Component**	**Bioactivity**	**References**
self-assembled NAs	HLJDD	50-200	BBR, BA	Antibacterial, anti-inflammatory	[21-25]
	HLWMD	281.2±18.9	Berberine, CGA	Anti-inflammatory	[26]
	Y-QY305	240.2±6.4	CGA, Loganin, Swertiamarine, Secoxyloganin, 4-Hydroxychalcone-4`-O-glucoside, Rutin, Isochlorogenic acid, Aldosecologanin, Glycyrrhizinic acide	Reduce cutaneous adverse reaction and diarrhea	[27]
	BHD	25-500	Mangiferin, neomangiferin	Antipyretic effect	[29, 30]
	MXSGD	78.82-712.40	Ephedrine, pseudoephedrine	Anti-influenza virus Antipyretic effects	[33-35]
	GGQLD	300-1000	BA, BBR, puerarin, glycyrrhizic acid	Antioxidant activity, hypoglycemic activity	[37, 38]
	GGD	<300	6-gingerol, glycyrrhizic acid	Antioxidant activity	[40]
	XBSD	104.53	Kukoamine B, mulberroside A, liquiritin	Enhancing oral bioavailability	[41]
	HGD	~100	Astragalus Polysaccharide, puerarin	Antithrombotic effect	[42]
	YB	86-726	Pseudoginseng	Blood coagulation	[48]
	CRD	200-500	BBR, palmatine	Promoting intestinal absorption	[52-54]
	GRD	100-800	Protein, glucose	Immunostimulant effect	[56, 57]
	Puerariae Lobatae Radix decoction	100-500	puerarin, daidzein, daidzin, genistein	Promoting intestinal absorption	[64, 65]
	Pseudostellariae Radix decoction	100-165	Protein	Promoting molecule absorption	[66, 67]
	IRD	57-300	Polysaccharides, protein	Antiviral activity	[72, 73]
	RCRR decoction	197.6	Small RNAs	Anti-fibrosis, anti-inflammatory	[76, 77]
Self-assembled CNAs	PTC	2-8	No small molecular component	Hemostatic effect	[80]
	CHC	1-5	No small molecular component	Hemostatic effect	[84]
	CJHC	2-11	No small molecular component	Hemostatic effect	[85]
	JMC	1-8	No small molecular component	Hemostatic and hepatoprotective activity	[86]
	SHC, SSC	1.29-6.87	No small molecular component	Hemostatic effect	[88, 89]
	CSH	1.4-2.8	No small molecular component	Hemostatic effect	[91]
	Phellodendri Chinensis Cortex Carbonisata	1.2-4.8	No small molecular component	Hemostatic effect	[92]
	TH-CD	2-5	No small molecular component	Repair the blood-brain barrier	[96]
	Crinis Carbonisatus	3.2-8.8	No small molecular component	Neuroprotective effect	[97]
	LJFC	1-10	No small molecular component	Anti-inflammation	[99]
	SFRC	2-3	-	Anti-inflammation, antioxidant activity	[102]
	Paeoniae Radix Alba Carbonisata	1.0-2.4	No small molecular component	Hepatoprotective effect	[103]
	PLRC	2-10	-	Anti-gout activity	[104, 105]
-	Aurantii Fructus Carbonisata	1.1-4.4	No small molecular component	Antihyperuricemic activity, anti-gouty arthritis activity	[106]
	Crataegi Fructus Carbonisata	1.3-5.6	No small molecular component	Hypoglycemic effect	[109]
	AAFC	6-10	No small molecular component	Anti-frostbite ability Antibacterial	[112, 113]
	Glycyrrhiza Radix Carbonisata	1-5	No small molecular component	Anti-ulcer activity	[115]
	Lycium barbarum	3.5	No small molecular component	Alleviating radiation-induced bone damage	[118]
	Coffee	2-5	CGA	Anti-tumor	[121]
	Green tea	3.07	No small molecular component	Antioxidant	[122]
Self-assembled EVNs	Fresh *Zingiber officinale*	150-400	6-Gingerol, 6-shogaol, phosphatidic acids, digalactosyldiacylglycerol	Anti-inflammatory, liver protection, antibacterial	[125-131]
	Fresh *Citrus limon*	30-100	-	Antioxidative effect, antitumor	[137-141]
	Fresh *Citrus paradise*	100-400	Quinic acid, oxalic acid, fructose	Anti-inflammation	[142]
	Fresh *Panax ginseng*	200-350	Ginsenoside Rg3, amiino acid, nucleotides, lipids acid	Immunomodulatory effect, antitumor	[149, 150]
	Fresh *Asparagus cochinchinensis*	90-200	Protein, lipids acid, RNAs	Antitumor	[152]
	Fresh *Dendropanax morbifera*	80-200	Protein	Antitumor	[153]
	Fresh Taraxacum officinale (T. officinal)	142.5	Protein, lipid	Preventing hypertension	[155]
	Fresh Morus nigra L. leaves	162.1	Lipids, flavonoid	Antitumor	[156]
	B. javanica fruits	104.6 ± 29.4	Lipids, proteins, miRNAs	Antitumor	[157]
	Fresh Rhizoma Drynariae roots	75.7±15.8	RNAs, proteins, lipids	Reversing osteoporosis	[158]
	Pueraria lobata	125.0±9.7	Lipids, proteins, miRNAs	Ameliorating mitochondrial dysfunction	[159]
	Fresh berries of Lycium barbarum L.	127.8±1.3	Saccharides, lipids	Inhibiting muscle atrophy	[160]
	Fresh Artemisia annua L.	106.8	Nucleic acids, proteins	Antitumor	[161]
	Fresh *Camellia sinensis*	131	Lipids, polyphenol, flavone	Antitumor	[162]
	Fresh *Vitis vinifera*	30-200	Protein, lipids acid	Anti-inflammation	[163-165]
	Fresh *Lentinula edodes*	100-140	Polysaccharide, lentinan, protein, lipids acid	Liver protection	[168]
	Fresh *Fragaria ananassa*	30-191	Vitamin C	Preventing oxidative stress	[169]
	Fresh *Brassica oleracea*	90-150	-	Anti-inflammation	[172]
-	Garlic	120	Proteins	Improving cancer immunotherapy	[173]

## LIST OF ABBREVIATIONS

AAFC: Artemisiae Argyi Folium Carbonisata
ADNVs: Artemisia-derived nanovesicles
BA: Baicalin
BBR: Berberine
BHD: Bai-Hu decoction
CGA: Chlorogenic acid
CL: Citrus limon L
CNAs: Carbonized nanoarchitectures
CP: Citrus paradise
CQDs: Food-derived carbon quantum dots
CrCi-CDs: Carbon dots derived from Crinis Carbonisatus
CRD: Coptidis Rhizoma decoction
CSH: Carbonisata Selaginellae Herba
ERα: Estrogen receptor-alpha
EVNs: Extracellular vesicle-like nanoparticles
GGQLD: Ge-Gen-Qin-Lian decoction
GR: Glycyrrhiza Radix et Rhizoma
GRD: Glycyrrhiza Radix et Rhizoma decoction
hBMSCs: Human bone marrow mesenchymal stem cells
HBP35: Hemin-binding protein 35
HGD: Huangqi Gegen decoction
HLJDD: Huang-Lian-Jie-Du decoction
HLWMD: Huanglian-Wumei decoction
IBS-D: Diarrhea-dominant Irritable Bowel Syndrome
IRD: Isatidis Radix decoction
JMC: Junci Medulla Carbonisata
JSX: Jiaosanxian
LJFC: Lonicerae Japonicae Flos Carbonisata
MXSGD: Ma-Xing-Shi-Gan decoction
NAs: Nanoaggregates
NLRP3: Pyrin domain-containing 3
NXD: Naoluo Xintong decoction
PDI: Polydispersity index
PG: Panax ginseng
PLRC-CNAs: Puerariae Lobatae Radix Carbonisata derived CNAs
PTC: Pollen Typhae Carbonisata
Pu-Exos: Pueraria lobata-derived exosomes
RCRR: Rhodiolae Crenulatae Radix et Rhizoma
RDNVs: Rhizoma Drynariae-derived nanovesicles
SFRC: Sophorae Flavescentis Radix Carbonisata
SHC: Schizonepetae Herba Carbonisata
SQDBD: Shi-Quan-Da-Bu decoction
SSC: Schizonepetae Spica Carbonisata
T-QY305: Qi Yin San Liang San decoction
TCM: Traditional Chinese medicine
TH-CDs: Taoren-Honghua carbon dots
TNBC: Triple-negative breast cancer
UCMS: Unpredictable chronic mild stress
XBSD: Xie-Bai-San decoction
YB: Yunnan Baiyao
ZRR: Zingiberis Rhizoma Recens
